# Evaluation of the Precision of Ancestry Inferences in South American Admixed Populations

**DOI:** 10.3389/fgene.2020.00966

**Published:** 2020-08-21

**Authors:** Vania Pereira, Roberta Santangelo, Claus Børsting, Torben Tvedebrink, Ana Paula F. Almeida, Elizeu F. Carvalho, Niels Morling, Leonor Gusmão

**Affiliations:** ^1^Section of Forensic Genetics, Department of Forensic Medicine, Faculty of Health and Medical Sciences, University of Copenhagen, Copenhagen, Denmark; ^2^Department of Mathematical Sciences, Aalborg University, Aalborg, Denmark; ^3^DNA Diagnostic Laboratory, State University of Rio de Janeiro, Rio de Janeiro, Brazil; ^4^Instituto de Investigação e Inovação em Saúde, i3S, Institute of Molecular Pathology and Immunology of the University of Porto (IPATIMUP), Porto, Portugal

**Keywords:** population stratification, ancestry informative marker, Brazil, biogeographical ancestry, population assignment

## Abstract

Ancestry informative markers (AIMs) are used in forensic genetics to infer biogeographical ancestry (BGA) of individuals and may also have a prominent role in future police and identification investigations. In the last few years, many studies have been published reporting new AIM sets. These sets include markers (usually around 100 or less) selected with different purposes and different population resolutions. Regardless of the ability of these sets to separate populations from different continents or regions, the uncertainty associated with the estimates provided by these panels and their capacity to accurately report the different ancestral contributions in individuals of admixed populations has rarely been investigated. This issue is addressed in this study by evaluating different AIM sets. Ancestry inference was carried out in admixed South American populations, both at population and individual levels. The results of ancestry inferences using AIM sets with different numbers of markers among admixed reference populations were compared. To evaluate the performance of the different ancestry panels at the individual level, expected and observed estimates among families and their offspring were compared, considering that (1) the apportionment of ancestry in the offspring should be closer to the average ancestry of the parents, and (2) full siblings should present similar ancestry values. The results obtained illustrate the importance of having a good balance/compromise between not only the number of markers and their ability to differentiate ancestral populations, but also a balanced differentiation among reference groups, to obtain more precise values of genetic ancestry. This work also highlights the importance of estimating errors associated with the use of a limited number of markers. We demonstrate that although these errors have a moderate effect at the population level, they may have an important impact at the individual level. Considering that many AIM-sets are being described for inferences at the individual level and not at the population level, e.g., in association studies or the determination of a suspect’s BGA, the results of this work point to the need of a more careful evaluation of the uncertainty associated with the ancestry estimates in admixed populations, when small AIM-sets are used.

## Introduction

Patterns of human genetic variation have been thoroughly investigated to unveil past events and disclose historical affinities among populations. Although most of the genetic variation can be observed within populations, a significant fraction can still be used to distinguish human populations, particularly from different continents. For that purpose, markers in a wide range of evolutionary rates and modes of inheritance have been used, showing clear differences between populations from Eurasia, sub-Saharan Africa, East Asia, America, and Oceania, even for small numbers of randomly selected markers.

In the last few years, many sets of Ancestry Informative Markers (AIMs) including SNPs and indels have been described to address individual ancestry or to detect diversity patterns between and within continental populations (Rosenberg et al., 2002; Nassir et al., 2009; Galanter et al., 2012; Pereira et al., 2012; Kidd et al., 2014; Phillips et al., 2014; Moriot et al., 2018; Cheung et al., 2019). To better capture the genetic differences among groups, these AIMs were selected to have large discrepancies in allele frequencies between populations. However, carefully selected markers are required to distinguish close population groups or to characterize continental fringe populations, which are often difficult to distinguish due to gene flow (Bulbul et al., 2016; Li et al., 2016; Yuasa et al., 2018; Pereira et al., 2019; Phillips et al., 2019).

The interest in studying AIMs is growing, and nowadays many DNA testing companies are offering online information on ancestry or genetic history to the average public in a fast and easy way. In forensic genetics, besides tracing back individual genealogies, AIMs can have a prominent role during the investigation phase of missing person cases and in the identification of crime perpetrators. AIMs are also used in clinical genetics, in case/control association studies, to avoid spurious associations due to population substructure (Marchini et al., 2004; Tian et al., 2008; Price et al., 2010).

The same AIM sets developed for human population genetics have also been used to investigate forensic cases. In this context, however, these sets are not usually utilized to question the continental ancestry of a sample contributor, but rather, the most likely population of origin of the DNA profile, i.e., the Biogeographical Ancestry (BGA) of a sample donor (Phillips et al., 2007; Rajeevan et al., 2012, 2020; Tvedebrink et al., 2017, 2018; Mogensen et al., 2020). However, inferring the most likely population of origin of an individual does not always provide direct information about its ancestry profile (and vice versa), namely in recently admixed populations. A set of markers that separates main population groups will not necessarily be the most adequate for determination of the apportionment of ancestry at an individual level, which requires several loci with large allele frequency differences among source populations.

Frequently used metrics proposed for AIM selection rely on the maximization of genetic distances or allele frequency differentials with the minimal number of markers (Pfaffelhuber et al., 2020). However, large genetic distances are usually associated with strong drift and/or selective pressure and, therefore, ancestry inferences or determination of the population of origin using few markers can be highly influenced by the correct definition of contributing or reference populations. The AIMs in use have always some degree of error associated when performing ancestry assignments, and one of the major challenges has been to select markers that minimize that error rate, increasing the accuracy of the studies or inferences.

In this work, we assessed ancestry with different sets of markers (46 indels developed for capillary electrophoresis and 165 SNPs included in the Precision ID Ancestry panel for massively parallel sequencing). Parent-offspring data from 65 families with mixed parentage were used. Since full-siblings have the same apportionment of common ancestry inherited from their parents, the most informative loci will be those presenting the smallest degree of deviation between the observed and expected ancestry proportions. Data on the genetic profiles of unrelated individuals from the Rio de Janeiro population considering the 210 AIMs are also reported.

We aimed to further investigate the factors that could cause differences in ancestry estimation and their impact when addressing ancestry at individual and population levels. Ultimately, these parameters can be used to understand how to achieve more accurate estimations, namely in populations harboring a trihybrid admixture from European, African, and Native American groups, which is typical for most South American populations.

## Materials and Methods

### Samples, Extraction of DNA and Quantification

Blood samples on FTA cards (Whatman Inc., Clifton, NJ, United States) were collected from 65 Brazilian families (with confirmed kinship) composed by mother, father, and two children (260 individuals in total), as well as from 84 unrelated Brazilian individuals. Informed consent was obtained from all participants included in the study. The project was approved by an ethical committee of the State University of Rio de Janeiro (CAAE: 0067.0.228.000-09).

DNA was extracted with the standard phenol-chloroform method. DNA extract concentration was measured using the InnoQuant HY kit (InnoGenomics) according to the manufacturer’s protocol or using the Qubit dsDNA High Sensitivity assay and the Qubit 2.0 Fluorometer (Invitrogen, Carlsbad, CA, United States) following the manufacturer’s instructions.

### Analysis of Ancestry Markers

The apportionment of the ancestry of each individual was investigated with different sets of AIMs. One set consisted of 46 indels selected to assess European, African, Asian, and Native American ancestries. The indels were amplified by PCR, and analyzed by capillary electrophoresis, according to Pereira et al. (2012). The individuals were also analyzed for 165 SNPs included in the Precision ID Ancestry panel (Thermo Fisher Scientific, Waltham, MA, United States) following the protocol recommended by the manufacturer. The DNA was sequenced using either the Ion PGM^TM^ or the Ion S5^TM^ platforms (Thermo Fisher Scientific). For the Ion PGM^TM^, each run contained 25 libraries (50 pM) loaded on an Ion 318^TM^ chip v2 (Thermo Fisher Scientific). For the Ion S5^TM^, 96 libraries (35–50 pM) were loaded on Ion 530^TM^ chips in each run (Thermo Fisher Scientific).

### Data Analysis

Allele calls for the 46 indels were considered for >50 RFUs for heterozygote individuals, and >100 RFUs for homozygote genotypes. For the 165 AIMs, allele calls were carried out following the same criteria as described in Santangelo et al. (2017).

The Precision ID Ancestry panel combines two published assays of 55 (Kidd et al., 2014) and 123 AIMs (Kosoy et al., 2009; Nassir et al., 2009), with 13 overlapping SNPs. Therefore, for ancestry inference analysis, the following five datasets were considered: 46 indels, 55 SNPs, 122 SNPs, 164 SNPs, and 210 markers (46 indels + 164 SNPs). The SNP rs10954737 was not included in the analyses, as it was not typed in all the African (AFR), European (EUR), and Native American (NAM) reference populations (hence, the analysis considered 164 SNPs instead of 165 SNPs).

Reference population data used in the analyses were available for all panels and consisted of 100 AFR, 100 EUR, and 47 NAM individuals. Data for 46 indels were retrieved from the 1000 Genomes database or were previously generated for HGDP-CEPH samples (Pereira et al., 2012). Genotypes for the same individuals for the 164 AIMs were kindly collected and provided by the Kidd Lab from publicly available data.

Allele frequencies, Hardy-Weinberg Equilibrium (HWE), genetic diversities, and pairwise *F*_ST_ genetic distances were calculated using the Arlequin v3.5.2.2 software (Excoffier and Lischer, 2010). HWE analysis was carried out using 1,000,000 Markov Chain Monte Carlo (MCMC) steps and 1,000,000 dememorization steps. Correction for multiple testing was done according to Bonferroni (1936). Statistical significance among genetic diversities was assessed with the *t*-test.

### Ancestry Inference

The distribution of NAM, EUR, and AFR genetic ancestry in each individual was estimated using the STRUCTURE v.2.3.4.21 software (Pritchard et al., 2000; Falush et al., 2003). The analysis was carried out using a burn-in period of 100,000 iterations, followed by 100,000 repetitions for the MCMC. The “admixture” and the “correlated allele frequencies” models were considered. Population information was used to assist clustering. Three assumed clusters (K) were considered in the analyses, and five independent runs were performed to verify the consistency of the results. The cluster membership coefficients of the five runs were combined using CLUMPP v.1.1.222 (Jakobsson and Rosenberg, 2007).

The apportionment of ancestry in each individual was plotted using the package “*plotrix”* developed for R software (R Core Team,, 2013). Statistical significance among average ancestry estimates was assessed with the z-score.

The combined individual ancestry values provided by CLUMPP were used to calculate all the parameters reported in this manuscript (average ancestry levels for the different datasets, absolute differences in ancestry among siblings and parents, and levels of variance reported in each component for all AIM sets).

### Population Assignment of Individuals

The assignment of individuals to a population of origin was assessed using the GenoGeographer software (Tvedebrink et al., 2018; Mogensen et al., 2020). In this analysis, the z-score was computed for each individual, considering AFR, EUR, NAM, and Rio de Janeiro as reference populations. The test considers the variance of the allele frequencies in the reference populations (Chakraborty et al., 1993), and the respective *p*-values are used to assess the most likely population of origin of the profile. The analyses were performed using a leave-one-out approach, excluding the individual tested from the reference dataset.

## Results

### Genetic Profile of the Rio de Janeiro Population for 210 AIMs

Data from 214 unrelated individuals (130 unrelated parents from 65 families, plus 84 additional unrelated individuals), living in Rio de Janeiro (Brazil), were used to calculate population descriptive statistics for 210 ancestry markers (164 SNPs + 46 indels). Three populations were used as reference – AFR, EUR, and NAM. Supplementary Table S1 contains detailed information on allele frequencies for these markers in Rio de Janeiro compared to the reference populations.

Three loci – rs1800414, rs3811801, and rs671 – were monomorphic in the sample from Rio de Janeiro. This result is in accordance with previous studies showing that these loci are only polymorphic in East Asian populations (Kidd et al., 2014; Pereira et al., 2017; Santangelo et al., 2017). As expected for an admixed population with NAM, AFR, and EUR ancestry, the remaining 207 markers were polymorphic in the Rio de Janeiro dataset. For the reference populations included in this study, the number of monomorphic loci was higher: 34 loci were monomorphic in the AFR reference population, 8 in the EUR sample, and 9 in the NAM group (Supplementary Table S1).

**FIGURE 1:**
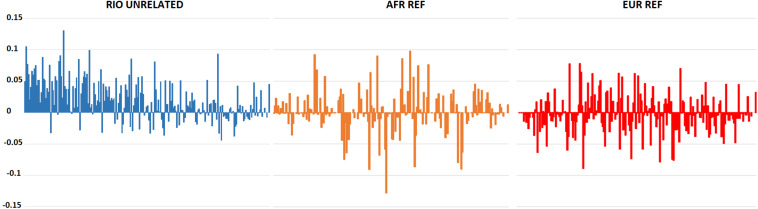
Differences between expected and observed heterozygosity values for the 210 AIMs in the Rio de Janeiro, AFR, and EUR reference samples. A positive value reflects an excess of homozygotes for the marker. Markers are presented in descending order of absolute allele frequency differences between AFR and EUR (more information on Supplementary Table S2).

Hardy-Weinberg Equilibrium was assessed for the 207 polymorphic markers in the Brazilian population. After correction for multiple tests, only rs6451722 presented a statistically significant deviation (*p*-value: 0.0002). This deviation was associated with an excess of observed homozygotes (63% compared to 50% expected under HWE), pointing to some degree of population stratification in Rio de Janeiro. Indeed, although statistically non-significant when applying the Bonferroni correction, 72% of the polymorphic loci showed lower observed heterozygosity values than expected in a population in HWE. The excess of homozygotes was higher for loci with greater differences in the allele frequencies between AFR and EUR populations, which are the main contributors to the current population of Rio de Janeiro (Figure 1). This general tendency for an excess of homozygosity was not observed in the AFR and EUR reference population samples.

The average genetic diversity was higher in the Brazilian sample (0.376 ± 0.179) than in any of the three continental references (AFR: 0.202 ± 0.098; EUR: 0.264 ± 0.127; NAM: 0.294 ± 0.142), reflecting the trihybrid origin of the Rio de Janeiro population. Differences in the genetic diversity values between Rio de Janeiro sample and all reference samples were statistically significant (*t*-test: *p*-value < 0.016).

**FIGURE 2:**
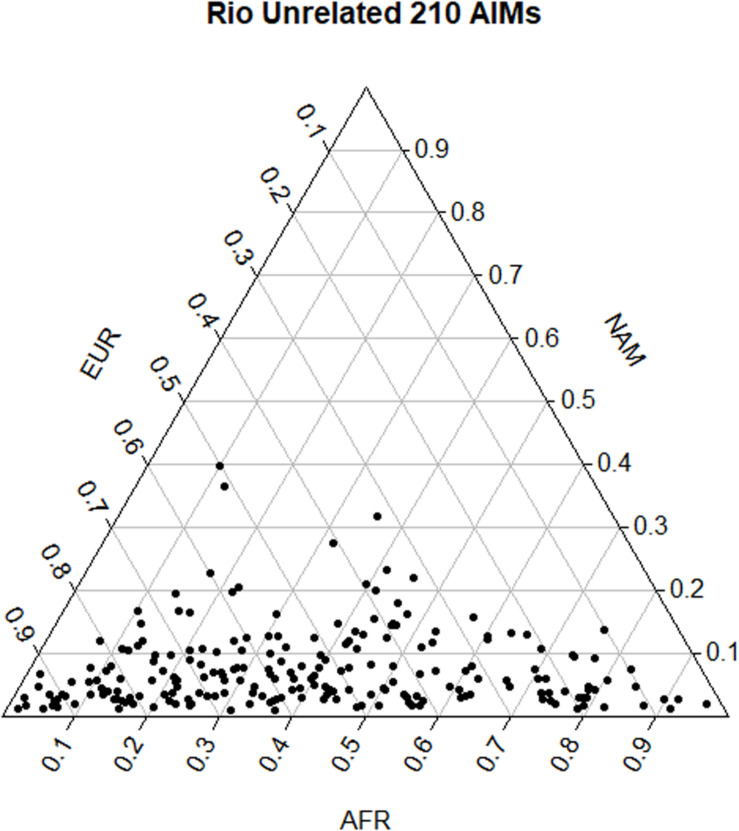
Triangular plot of the q-matrices generated in STRUCTURE and combined in CLUMPP, considering the distribution of the three ancestry components in each individual.

Pairwise *F*_ST_ values among populations showed a smaller differentiation between the Brazilian dataset and the EUR reference (*F*_ST_ = 0.113) than with AFR (*F*_ST_ = 0.212) and NAM (*F*_ST_ = 0.314), which is in accordance with the distribution of ancestry proportions in the Brazilian sample. STRUCTURE results showed that the EUR component was the one with the highest contribution (54.0%), followed by the AFR (38.5%), and the NAM (7.5%) components. The apportionment of ancestry in each individual is plotted in Figure 2. The wide dispersal of individuals across the plot (mostly along the AFR and EUR axes) is consistent with a great intrapopulation variation, compatible with recent admixture events and/or a certain degree of population substructure.

**FIGURE 3:**
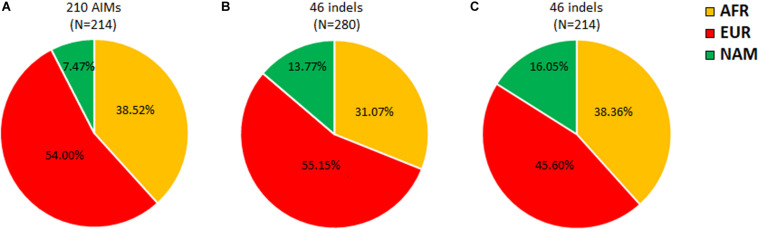
Apportionment of ancestry in Rio de Janeiro based on 210 AIMs **(A)**, 46 indels from Manta et al., 2013 **(B)**, and the current dataset **(C)**.

A previous study using the same 46 ancestry informative indels as in this work, but in 280 individuals from Rio de Janeiro, reported slightly different ancestry proportions (Manta et al., 2013; Figure 3B). Furthermore, the difference between the NAM proportions in both studies (Figures 3A,B) was statistically significant (z-score *p*-value = 0.0271). Since the 46 indels are a subset of the 210 AIMs analyzed here, we recalculated the average ancestry values for our sample of 214 individuals based on the 46 indels alone (Figure 3C).

Comparing the results obtained in the two population samples from Rio de Janeiro for the 46 indels (Figures 3B,C), higher AFR and NAM contributions were detected in this study. Although differences in these two components were not high enough to be statistically significant (AFR z-score *p*-value = 0.09102; NAM z-score *p*-value = 0.4902), a statistically significant decrease of 9.6% (z-score *p*-value = 0.03486) was found in the EUR component. This variation observed for the same AIM panel could be related to the sampling in both studies. Manta et al. (2013) investigated randomly selected unrelated individuals born in the metropolitan region of Rio de Janeiro. In the current study, the samples were collected from paternity cases from Rio de Janeiro that also include surrounding areas outside the metropolis. Variation in the ancestry contributions across Rio de Janeiro has been reported by others (Almeida et al., 2017). A previous study that evaluated ancestry inference when using different sampling cohorts from the Rio de Janeiro population reported increased AFR and decreased EUR contributions outside the metropolitan region (Almeida et al., 2017). This sampling effect is also observed in this work.

Differences in the ancestry components were not only observed between the two studies but also when comparing the same individuals analyzed in this work for the 210 and 46 markers (Figures 3A,C). Compared to the complete AIM set, the 46 indels reported increased NAM and decreased EUR ancestry proportions. The difference in the NAM component was statistically significant (z-score *p*-value = 0.00578).

In the following sections, we intended to investigate the factors that may influence these differences in ancestry estimation and their impact when addressing ancestry at the population and individual levels. Ultimately, we aimed to disclose and compare the effect of the parameters that most influence ancestry determination. This will help to understand how to achieve more accurate estimates, particularly in populations harboring a trihybrid admixture from EUR, AFR, and NAM groups, like the Brazilian population.

### Factors Influencing Ancestry Estimations at the Population Level

Although ancestry estimates can be deduced from full genomes or genome-wide studies, the overall ancestry at both population and individual levels is most often calculated based on a certain number of genetic markers showing low discrepancies to the genome-wide results (e.g., Galanter et al., 2012; Santos et al., 2016). Since just a limited portion of the entire genome is analyzed, the accuracy of the results relies on the type and number of selected markers. Loci with low variation among the source populations will tend to give poor ancestry estimates. In these cases, an overestimation of the less represented ancestry components at the expense of those most represented in the population is expected, as seen previously for Rio de Janeiro (Figures 3A,C).

Similarly, even if the markers are highly informative, a balanced discriminatory power between reference populations is also required. As shown in Galanter et al. (2012), a lower mean locus-specific branch length for European ancestry results in an underestimation of this component in MXL and PUR subjects. The same was observed for the AFR ancestry in that study. Besides these factors, the number of markers may also play a role when addressing ancestry, since a low number of autosomal loci, even if unlinked, may lead to stochastic variations in the representativeness of the different ancestors.

To explore this issue further, we compared the ancestry estimates obtained when using different AIM sets in several American admixed populations.

#### Ancestry Estimates in Rio de Janeiro Using Different Panels

Average values of ancestry among the unrelated samples from Rio de Janeiro were calculated after dividing the data into several datasets. The Precision ID Ancestry panel combines two ancestry sets: the 55 SNPs selected by the Kidd lab (Kidd et al., 2014), and 123 out of the 128 SNPs selected by the Seldin lab (Kosoy et al., 2009; Nassir et al., 2009). The strategies for marker selection of the panels were slightly different. The 55 SNP panel was made to contain few markers to identify the BGA of an unknown sample. The SNPs are representative of diverse geographical regions, and the selection process included pairwise comparisons of reference populations to select those markers with the largest allele frequency differences. The final set was balanced between population groups so that the geographic regions could be distinguishable with the same level of robustness (Kidd et al., 2014). The strategy for the development of the 128 SNP panel from the Seldin lab was to include markers with large allele frequency differences among European, Sub-Saharan African, American, and East Asian groups (Kosoy et al., 2009; Nassir et al., 2009).

**FIGURE 4:**
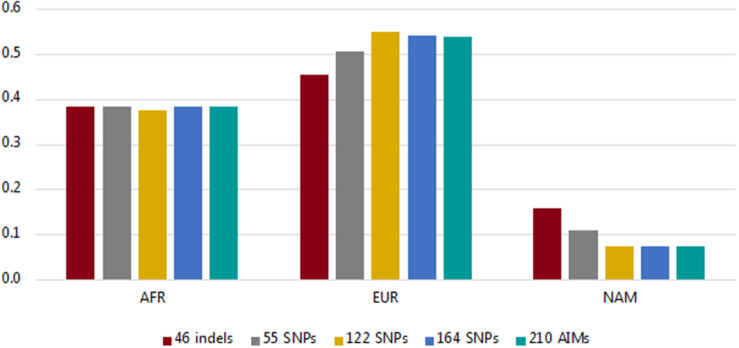
Average ancestry proportions per component for the Rio de Janeiro sample reported by the five AIM sets considered.

Using the information from the unrelated individuals (*N* = 214), we compared the average ancestry proportions per component reported by the five panels (46 indels, 55 SNPs, 122 SNPs, 164 SNPs, and the total dataset of 210 AIMs) to evaluate the level of variation among them (Figure 4; more information on the average, range, and variance of the ancestry values reported for each panel is presented in Supplementary Table S3).

The values for the AFR component were similar for all sets of markers (values varied from 0.3755 for 122 SNPs to 0.3852 for the 210 AIMs). The variation was higher for the EUR and NAM components, which represent the highest and lowest ancestry proportions, respectively (discussed in more detail below).

A previous study that compared ancestry inferences in admixed samples from Brazil and Colombia using 30 ancestry and 30 identity indel-markers showed that the proportions of each component in a trihybrid population always tended to be equally divided for human identity markers that were not optimal for discrimination of ancestry (Aquino et al., 2015). Therefore, when the true ancestry proportions were not captured by the selected markers, for *K* = 3 there was a tendency to overestimate ancestry to values closer to 33%, and vice-versa: values above 33% tended to be underestimated.

In the studied population, the AFR ancestry component is close to 33% (Figure 4). As stated, smaller ancestry differences might not be captured at this level, regardless of the panel used, which may be why no significant difference was observed in the AFR estimates with the different AIM sets.

More variation was observed for the EUR and NAM components (Figure 4 and Supplementary Table S3). The EUR component was smaller when the samples were analyzed for the 46 indels, and conversely, this was the panel reporting the highest value of the NAM component (Figure 4 and Supplementary Table S3). As stated previously, if the panels provide low levels of population differentiation, a tendency to underestimate the major ancestry component (in this case, EUR) and to overestimate the minor component (NAM) would be expected, as seen for the 46 indel panel. Although to a lesser extent, this tendency was also observed in the 55 SNP set.

#### Ancestry Estimates in American Admixed Populations From the 1000 Genomes

**FIGURE 5:**
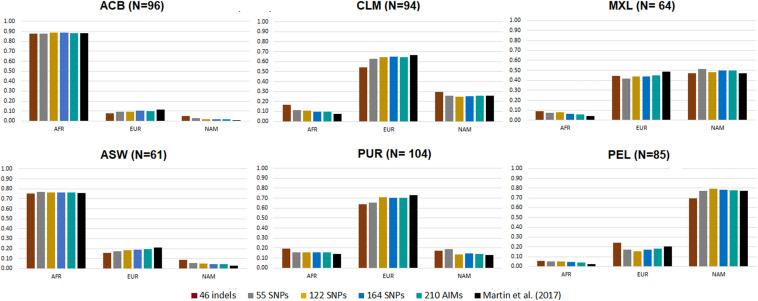
Average ancestry proportions for the five AIM panels in six American admixed populations from the 1000 Genomes database. Reference values based on genome-wide SNP data are represented in black (Martin et al., 2017).

African, European, and Native American ancestry components were estimated for the previously defined AIM sets using data from six American admixed populations included in the 1000 Genomes Project (phase 3): African Caribbean in Barbados (ACB); Americans of African ancestry in Southwest United States (ASW); Colombians from Medellin, Colombia (CLM); Mexican Ancestry from Los Angeles, United States (MXL); Peruvians from Lima, Peru (PEL); and Puerto Ricans from Puerto Rico (PUR). The results for each panel of AIMs were compared to the ancestry estimates based on common genome-wide SNPs (Martin et al., 2017; Figure 5; more information on the average, range, and variance of the ancestry values reported for each panel is presented in Supplementary Table S4). The triangular plots of the individual q-matrices generated in STRUCTURE for the six populations based on 210 AIMs are presented in Supplementary Figure S1.

The six admixed populations could be divided into four groups: Populations with mainly AFR ancestry (ACB and ASW), populations with mainly EUR ancestry (CLM and PUR), a population with mainly NAM ancestry (PEL), and a population with similar proportions of EUR and NAM ancestries (MXL).

Considering the estimates for genome-wide data as reference, the ancestry values reported by the 46 indels overestimated the minor components and underestimated the major ancestry components for all American populations, except for populations with high AFR ancestry (ACB and ASW), similarly to the observation in the population from Rio de Janeiro (Figure 4). For the other AIM panels, there was a small underestimation of the EUR component compared to the values obtained with the genome-wide SNPs (EUR reference values varying from 11.7 to 73.2%; AFR varying from 2.5 to 88%). In contrast, the NAM component was overestimated (NAM reference values between 0.3 and 77.3%).

However, it is worth noting the relatively low variation among all sets in most populations. Most estimates fell within the interval defined by one standard deviation of the average reference values (Martin et al., 2017; Supplementary Table S4). Few cases were the exception, namely: the AFR component in MXL, for 46 indels, 55 SNPs, and 122 SNPs; the EUR component in PUR, for the 46 indels; the NAM component in PUR, for 46 indels and 55 SNPs; and the NAM component in ACB, for all sets.

**FIGURE 6:**
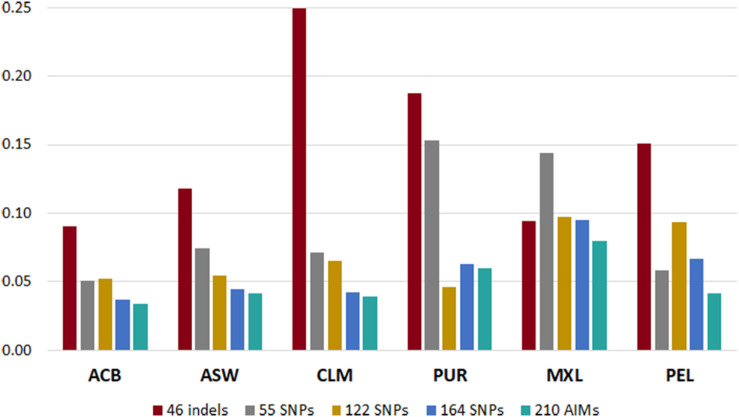
Sum of deviations from the reference values (Martin et al., 2017) reported by each panel in the six admixed American populations from the 1000 Genomes database.

To understand which panel presented more variation compared to the reference values based on the genome-wide SNPs (Martin et al., 2017), we calculated the sum of all the absolute deviations from the reference values for the five panels in each population (Figure 6). Different trends could be seen for each panel, depending on the ancestry profile of the population.

In all populations, the 210 AIM set had the smallest accumulated error for the three continental components. The 46 indels performed worst in most populations, but it presented smaller deviations than the 55, 122, and 164 SNP panels in the MXL population. This population had similar proportions of EUR and NAM ancestries (Figure 5). For the populations with lowest NAM ancestry, the combination of 46 indels and 164 SNPs did not substantially improve the accuracy of the estimates compared to the 164 SNP panel alone.

However, for populations with high proportions of NAM ancestry (MXL – 40.6% and PEL – 77.6%), the inclusion of the 46 indels improved the estimates obtained with the 164 SNPs of the Precision ID Ancestry panel.

The type of errors seen for the 46 indels can be explained by the low number of markers and/or low *F*_ST_ values among the three populations. As for the remaining panels, the systematic biases were more likely due to an unbalanced genetic differentiation among populations, with EUR-NAM showing the lowest *F*_ST_ value (discussed in more detail below).

#### Number of Markers and the Genetic Differentiation of the Reference Populations

**FIGURE 7:**
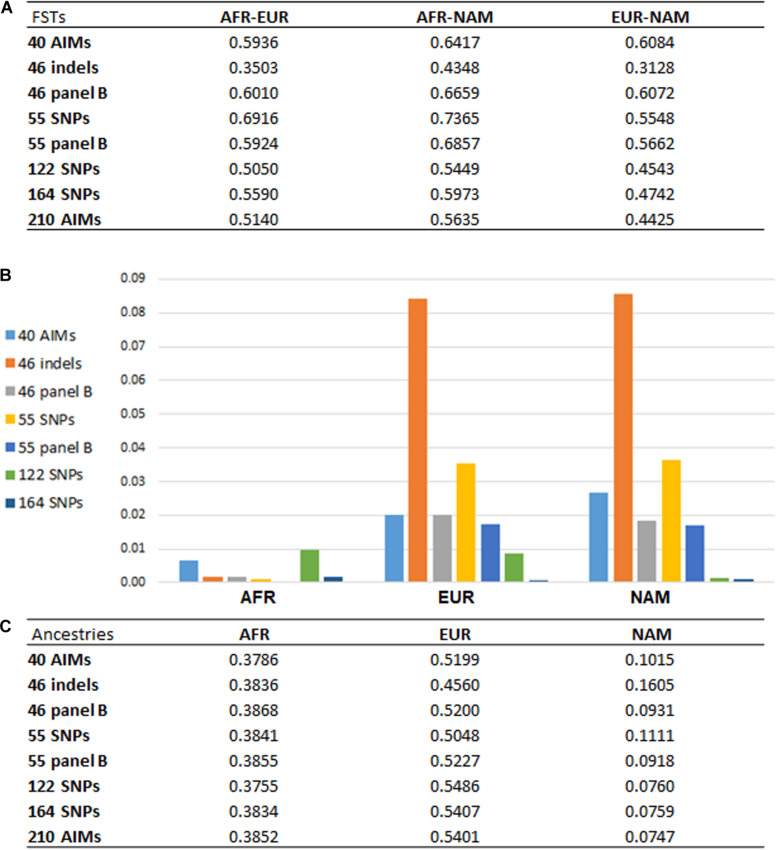
**(A)** pairwise FST values among reference populations (AFR, *N* = 100; EUR, *N* = 100; NAM, *N* = 47) based on the different AIM panels; **(B)** absolute values of the differences between the average ancestries reported for each panel compared to the 210 AIMs; **(C)** average ancestry values per component and per panel for unrelated individuals.

From the results obtained for the different AIM-sets, it can be seen that the number of loci and their capacity to differentiate source populations influence the accuracy of the ancestry estimations. With a higher number of loci, the variations associated with the estimations were smaller, as seen for example in the inferences provided by the 122, 164, and 210 AIM panels (Figures 4, 5). Apart from the variation in the number of loci, the five panels presented different pairwise *F*_ST_ values among the three reference populations (Figure 7A).

This leads to the question of whether results of ancestry inferences are more dependent on the number of markers included in an AIMs panel than the combined population differentiation these markers provide, or if they are equally dependent on both?

To address this issue, we returned to the global set of 210 AIMs, and defined three additional AIM panels based on different selection strategies (more details on Supplementary Information 1):

(a) two new panels with 46 and 55 AIMs (named 46 panel B and 55 panel B), where we aimed to maintain the same number of markers but selected those that would have the highest and most balanced pairwise *F*_ST_s among all population groups. The distances among EUR-NAM were given preference since they had the smallest distances in the original panels;

(b) a new panel with a small number of markers (40 AIMs), but the emphasis was now on the selection of the combination of markers that produced smaller differences between the *F*_ST_s among the reference groups (i.e., same levels of differentiation between AFR-EUR, AFR-NAM, and EUR-NAM).

Figure 7 presents the pairwise *F*_ST_s among reference populations obtained with each panel (Figure 7A), the average ancestry proportions reported for the 214 unrelated individuals (Figure 7C), and their absolute differences compared to the estimates provided by the 210 AIMs (Figure 7B).

Looking at the pairwise *F*_ST_ values (Figure 7A), we observed that the number of markers is not the only factor responsible for the differences previously reported in the ancestry estimations (Figure 7C). Panels with the same number of markers presented different magnitudes of *F*_ST._ Compared to the 46 indels, the 46 panel B had greater *F*_ST_s among the three population groups, and they were similar to the *F*_ST_s for larger panels. In Figure 7B, the new 46 panel B has much smaller deviations from the ancestry values obtained with the total set of 210 AIMs, and it appears to perform better than the 55 SNP panel, which has less balanced pairwise *F*_ST_s (Figure 7A).

For the two panels with 55 markers, smaller *F*_ST_ values were obtained for AFR-EUR and AFR-NAM. For EUR-NAM, which was the genetic distance that was prioritized upon selection of these markers, the *F*_ST_ was slightly higher. The 55 panel B also showed smaller differences compared to the 55 SNP set (Figure 7B), probably due to more balanced pairwise *F*_ST_s among the source populations.

Compared to the 46 indel and 55 SNP sets, the average ancestry values obtained with the 40 AIMs were overall closer to those reported for the 210 panel (Figure 7C); differences ranged from 0.0077 in the AFR component to 0.0277 in the NAM component. As expected from the selection criteria, the *F*_ST_ values based on the 40 AIMs were higher than those obtained for other less balanced panels, or panels with a higher number of markers (Figure 7A). However, when the number of markers included in the panel increases to 122 or 164, the ancestry estimates were closer to those obtained for the full set, with no significant variation observed between 164 AIMs and the total set of 210 AIMs. A similar trend was observed when comparing the performance of the three newly selected sets in the six American populations from the 1000 Genomes project (Supplementary Figure S2). However, the errors associated to each panel showed a variation that depends on the ancestry profile of the populations.

The results highlight that a balanced population differentiation among the reference groups also plays an important role in the accuracy of the ancestry estimations, especially for small sets of 40–55 SNPs. Large AIM sets (e.g., the 164 AIMs), result in smaller variation in the ancestry estimates even if these panels had slightly lower and less-balanced *F*_ST_s.

### Factors Influencing Ancestry Estimations at the Individual Level

As illustrated above, differences in ancestry estimates are expected when using different groups of AIMs. These differences can be due to the poor performance of the markers to differentiate ancestry components. In this case, there will be a directional bias in the estimations, and some ancestry components will tend to be overestimated at both the individual and population level. However, if the differences are not related to the marker performance, but with the (low) number of markers used, it is expected that the differences in population genetic statistics will be random. These variations will have a much smaller effect in larger population samples than at the individual level.

#### Individual Ancestry Estimates Using Different Panels

To investigate differences in ancestry estimates at the individual level, we plotted the pairwise comparisons between the panels of 46 indels, 55 SNPs, and 122 SNPs.

**FIGURE 8:**
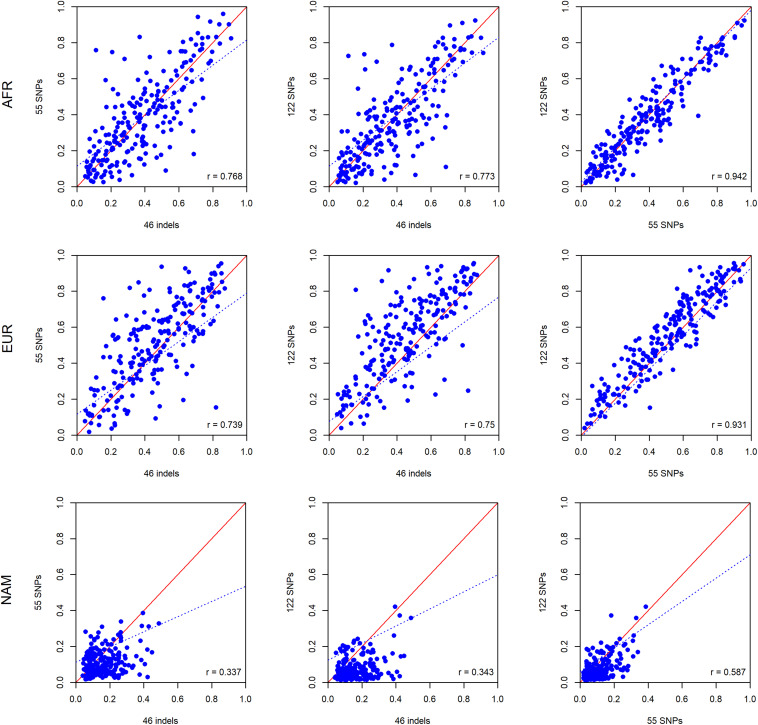
Pairwise comparisons between ancestry estimates provided by the panels with 46 indels, 55 SNPs, and 122 SNPs among the unrelated individuals from the Rio de Janeiro population. The respective *r*-values are indicated in the figures, and the tendency line is represented as a blue dashed line. The red solid line indicates the perfect agreement between two AIM panels.

As can be seen in Figure 8, there are large differences at the individual level when the results of the three panels are compared. The results are in accordance with the ancestry estimates at the population level, with the NAM component showing the worst results. The highest levels of correlation and agreement among comparisons were found between the 55 SNP and 122 SNP panels for the AFR and EUR components (*r* = 0.942 and *r* = 0.931, respectively). The correlation was lower for the NAM component in all pairwise comparisons (*r* ≤ 0.585).

The results for the six American admixed populations of the 1000 Genomes Project showed a similar trend, with the 55 SNP and 122 SNP panels presenting the highest levels of correlation and agreement among comparisons. However, the component with the largest differences varied among populations. The most extreme disagreement among the three panels was obtained for the NAM component in ACB and for the AFR component in MXL (Supplementary Figures S3–S8).

#### Comparison of Average Ancestry Proportions in Parents vs. Offspring

Families with parents and their offsprings are excellent proxies to study the variation of ancestry estimations at the individual level. To this end, data from 65 families (mother, father, and two offsprings) with confirmed kinship were investigated (*N* = 260). The estimates reported by the five panels (46 indels, 55 SNPs, 122 SNPs, 164 SNPs, and 210 AIMs) were compared once again considering that: (1) the apportionment of ancestry in the offspring should be close to the average ancestry of the parents, and (2) full siblings should present very similar ancestry values for a set of unlinked markers.

In this context, the most informative group of loci will be the one presenting the smallest difference in ancestry between the siblings and their parents.

**FIGURE 9:**
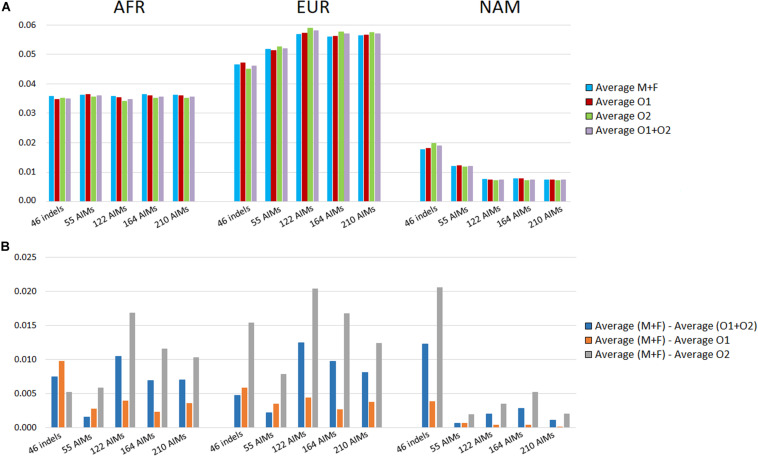
**(A)** Average ancestry values per component reported by the five panels in parents (mother and father, M + F) and the offspring (offspring 1, offspring 2, and O1 + O2); **(B)** absolute differences between the average of the parents and: the average of both offspring (blue), the average of the offspring 1 (orange), and the average of the offspring 2 (light gray).

We looked at the variation in the average ancestry proportions provided by STRUCTURE for the datasets defined by the parents (mothers and fathers, M + F) and the offspring (O1, O2, and O1 + O2). The average values and their absolute differences are presented in Figure 9.

In theory, the average ancestry proportions in these four groups should be similar, but differences were observed between the estimates. The largest difference between datasets was 2% for the EUR component estimated by the 122 AIMs, and for the NAM, when using 46 indels. The variation in the ancestry proportions was observed regardless of the number of markers included in the panel. The set of 55 SNPs had the lowest variation (all values were below 0.078%).

The analyses were based on a limited number of loci and a random variation was expected that depended on the numbers of markers and samples analyzed. However, there was no clear correlation between the number of markers and the differences in the variation observed between parents and offspring subsamples. We can, therefore, conclude that if there is a drift effect at the individual level, this is not reflected at the population level for the number of samples analyzed here. A directional bias could also influence the differences observed within each panel. For instance, an approximation of ancestry components to a certain value will result in a smaller difference among individuals.

**FIGURE 10:**
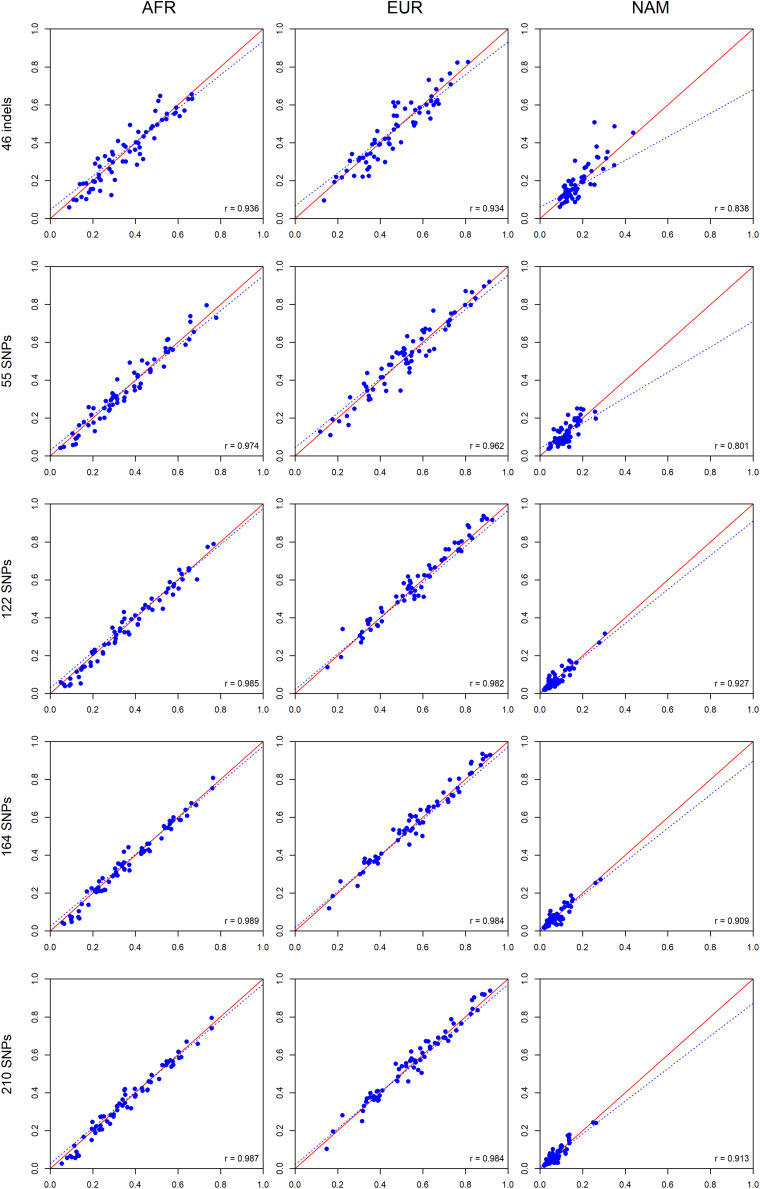
Pairwise comparisons between the average ancestry estimates of the parents and the average estimates of their offsprings based on five AIM sets. The respective *r*-values are indicated in the figures, and the tendency line is represented as a blue dashed line. The red solid line indicates the perfect agreement between two AIM panels.

To investigate the expected variation of ancestry estimates at the individual level, we compared the average ancestry of the parents and offsprings for each component (Figure 10).

A high positive correlation was obtained for all AIM sets, and the values were closer to *r* = 1 when the number of markers was increased. For the AFR component, the highest correlation was observed for the 164 SNPs (*r* = 0.989). For the EUR component, the 164 SNPs and total AIM set of 210 markers presented the highest values (*r* = 0.984). For the NAM component, the highest value (*r* = 0.927) was reported for the 122 SNPs.

**FIGURE 11:**
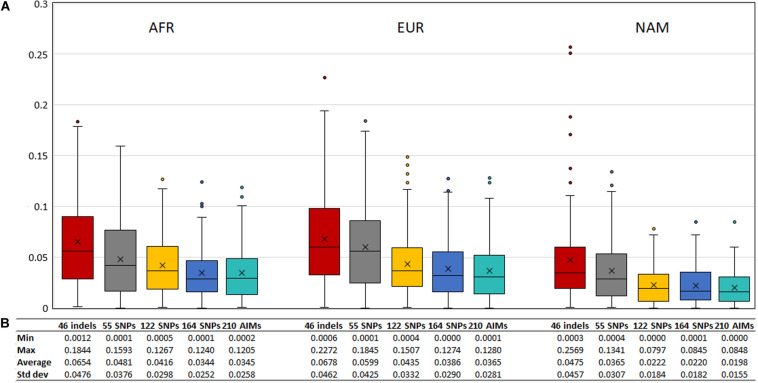
**(A)** Box and whisker plot of the absolute differences between the ancestry estimated in the 130 offsprings and the average ancestry of the parents with the five AIM sets. The box represents the interquartile range between the first and the third quartiles. Line in the box displays the median value and the x displays the mean value. Whiskers define 1.5 times the interquartile range. **(B)** The minimum, maximum, average values, and the respective standard deviations are also presented for each panel and component.

To evaluate the agreement between the observed ancestries for each offspring and the expected values given by the average ancestry of the parents, we calculated the absolute differences of these values, shown in Figure 11. The differences decreased when a higher number of markers was used.

Although the differences between the average ancestry estimated in parents vs. offspring were lowest for the 55 SNPs at the population level (Figure 9B), the full set of 210 AIMs produced the smallest variation at the individual level. Moreover, the addition of the 46 Indels to the 164 SNPs had the highest effect in the NAM component, which is in accordance with what was observed for the estimates obtained at the population level (Figure 5 – Section “Ancestry Estimates in American Admixed Populations From the 1000 Genomes”).

#### Comparison of Ancestry Estimates Among Sibling Pairs

**FIGURE 12:**
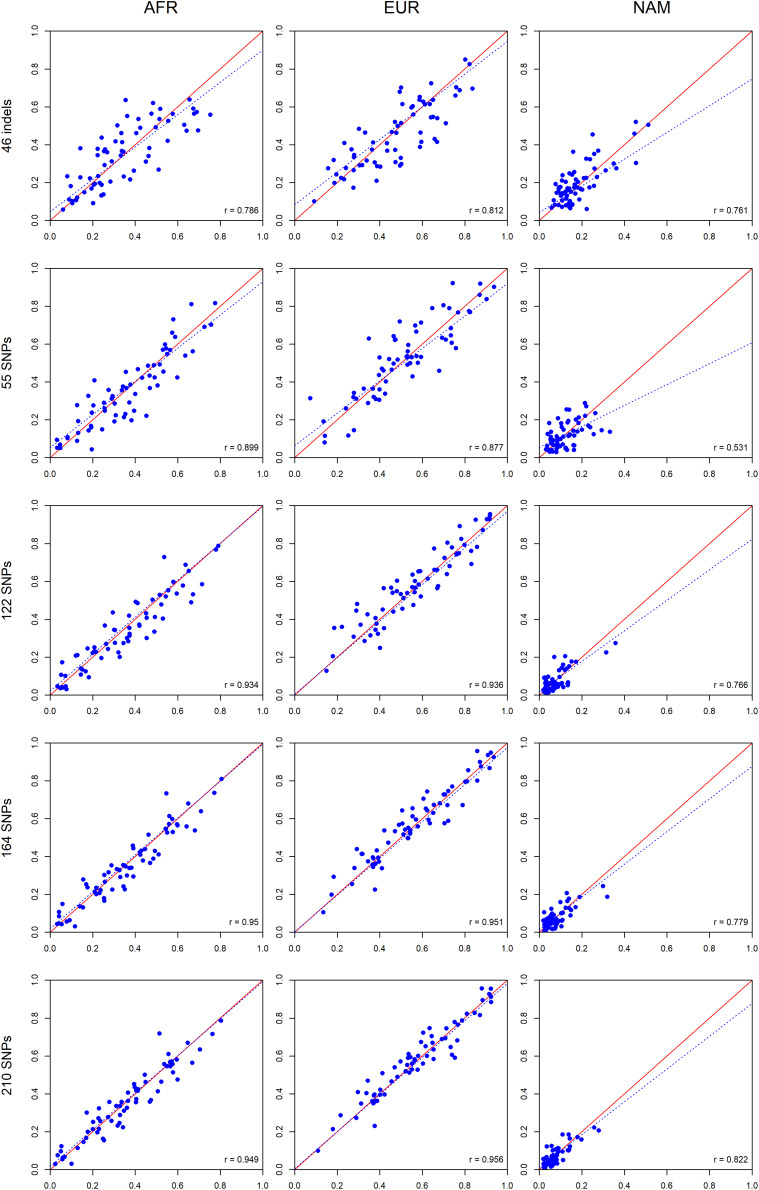
Comparisons between the ancestry estimates for siblings pairs based on the five AIM panel. The respective *r*-values are indicated in the figures, and the tendency line is represented as a blue dashed line. The red solid line indicates the perfect agreement between two AIM panels.

A further comparison was performed between siblings (Figure 12) based on the assumption that siblings should have identical ancestry components from the three continental sources when accessed by a large enough number of well balanced AIMs.

As for the comparisons between parents and offsprings, a high correlation was also observed between the ancestry proportions of siblings. The smallest *r*-value was 0.531 for the 55 SNPs in NAM; all other correlations were above 0.761 (Figure 12). There was an overall tendency of increased correlation with an increase in the number of markers.

**FIGURE 13:**
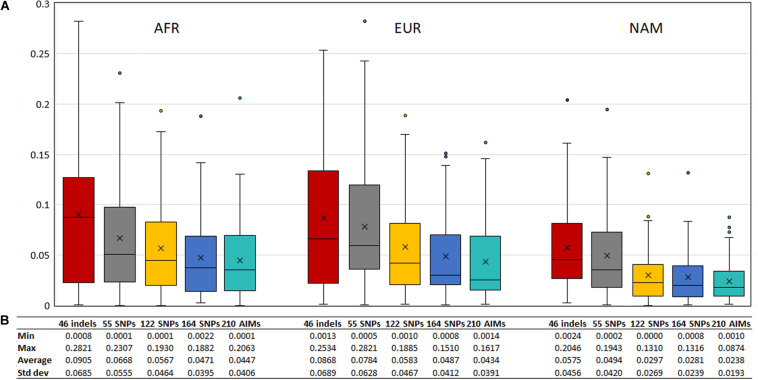
**(A)** Box and whiskers plot of the absolute differences between the ancestry values obtained for siblings based on results with the five AIM sets. See Figure 11 for the explanation of the Box and whiskers plot. **(B)** The minimum, maximum, average values, and the respective standard deviations are also presented for each panel and component.

The concordance between the ancestries of the siblings was measured by calculating the absolute differences observed (Figure 13). Again, smaller differences among siblings were obtained with increasing numbers of markers. The 210 AIM panel had the smallest deviation in ancestry estimations among siblings (Figure 13).

For all AIM sets, both correlation and concordance were higher between parents vs. offspring than between siblings.

### Inferences on Biogeographical Ancestry (BGA)

The five previously defined AIM sets were used for prediction of the biogeographical origin of the profiles from Rio de Janeiro, considering four reference populations: AFR, EUR, NAM, and Rio de Janeiro. A z-score test was applied to the 214 unrelated individuals and to each offspring of the 65 sibling pairs from Rio de Janeiro, to assess whether one (or more) of the four reference populations was accepted as a potential population of origin of each AIM profile. This test was performed using the approach described in Tvedebrink et al. (2018).

#### Biogeographical Ancestry Inferences in Rio de Janeiro Using Different Panels

The accuracy of BGA inferences for the five AIM sets was estimated considering AFR, EUR, NAM, and Rio de Janeiro as the potential source populations. To this end, for all AIM sets, we evaluated the proportion of individuals that were classified as “Rejected” (none of the four reference populations was defined as a possible population of origin of the profile; z-score > 1.64, *p*-value < 0.05) or “Accepted” (at least one of the four reference populations was defined as a possible population of origin of the profile; z-score ≤ 1.64, *p*-value ≥ 0.05).

Among the cases defined as “Accepted,” it was also calculated (1) the proportion of “concordant” assignments (individuals accepted in the true population of origin or, when accepted in more than one population, a significant higher likelihood was obtained for the true population of origin), (2) “discordant” (individuals accepted in a population that was not the true population of origin, or accepted in the true population but with a significantly lower likelihood than in another), and (3) “ambiguous” (individuals accepted in more than one population with non-significantly different likelihoods).

The results in Table 1 show that there is a relatively high rate of rejection, depending on the population and the panel considered. The highest values were found for the Rio de Janeiro samples. In this population, the percentage of samples rejected increased for larger panels, reaching 31% for the 210 AIMs. Except for the AFR, the 46 Indels showed discordant results that reach 21% in Rio de Janeiro. Although with high percentage of rejection, larger panels show higher percentage of concordant profiles. However, even for the AIM sets with high concordance, there is still 9% of individuals being assigned to the wrong population.

**TABLE 1:** Results of BGA inferences for five AIM panels, considering AFR, EUR, NAM, and Rio de Janeiro populations.

		Accepted			
	Panel	Ambiguous	Concordant	Discordant	Rejected
	46 indels	0 (0.00%)	93 (100.00%)	0 (0.00%)	7 (7.00%)
AFR	55 SNPs	0 (0.00%)	89 (100.00%)	0 (0.00%)	11 (11.00%)
	122 SNPs	0 (0.00%)	92 (100.00%)	0 (0.00%)	8 (8.00%)
	164 SNPs	0 (0.00%)	91 (100.00%)	0 (0.00%)	9 (9.00%)
	210 AIMs	0 (0.00%)	91 (100.00%)	0 (0.00%)	9 (9.00%)
EUR	46 indels	2 (2.02%)	94 (94.95%)	3 (3.03%)	1 (1.00%)
	55 SNPs	0 (0.00%)	90 (100.00%)	0 (0.00%)	10 (10.00%)
	122 SNPs	0 (0.00%)	97 (100.00%)	0 (0.00%)	3 (3.00%)
	164 SNPs	0 (0.00%)	96 (100.00%)	0 (0.00%)	4 (4.00%)
	210 AIMs	0 (0.00%)	97 (100.00%)	0 (0.00%)	3 (3.00%)
NAM	46 indels	0 (0.00%)	41 (97.62%)	1 (2.38%)	5 (10.64%)
	55 SNPs	0 (0.00%)	39 (100.00%)	0 (0.00%)	8 (17.02%)
	122 SNPs	0 (0.00%)	36 (100.00%)	0 (0.00%)	11 (23.40%)
	164 SNPs	0 (0.00%)	34 (100.00%)	0 (0.00%)	13 (27.66%)
	210 AIMs	0 (0.00%)	33 (100.00%)	0 (0.00%)	14 (29.79%)
Rio de Janeiro	46 indels	8 (4.60%)	129 (74.14%)	37 (21.26%)	40 (18.69%)
	55 SNPs	0 (0.00%)	151 (90.96%)	15 (9.04%)	48 (22.43%)
	122 SNPs	1 (0.63%)	134 (84.81%)	23 (14.56%)	56 (26.17%)
	164 SNPs	0 (0.00%)	137 (91.95%)	12 (8.05%)	65 (30.37%)
	210 AIMs	0 (0.00%)	135 (91.22%)	13 (8.78%)	66 (30.84%)

*“Rejected” – none of the four reference populations was defined as a possible population of origin of the profile; z-score > 1.64, *p*-value < 0.05; “Accepted” – at least one of the four reference populations was defined as a possible population of origin of the profile; z-score ≤ 1.64, *p*-value ≥ 0.05; “Ambiguous” – accepted in more than one population with non-significantly different likelihoods; “Concordant” – accepted in the true population of origin or, when accepted in more than one population, a significant higher likelihood was obtained for the true population of origin; “Discordant” – accepted in a population that was not the true population of origin, or accepted in the true population but with a significantly lower likelihood than in another.*

The final proportion of all cases that were accepted in the true population with significant higher likelihood was only 63% for the largest panel (135 individuals out of the 214). Taking together both sensitivity and specificity (concordant results), the 55 SNPs presented the highest rate of assignment of individuals in the true population of origin (71%).

The discordant assignments were mainly due to the low discrimination between EUR and Rio de Janeiro. Comparing the z-scores obtained with the different panels (Supplementary Figure S9) it is possible to see an overlap of the z-scores for the EUR and Rio de Janeiro samples when considering EUR as the population of origin.

#### Comparison of Biogeographical Ancestry Estimates Between Sibling Pairs

To compare the results among the two siblings, we investigated (1) how many sibling pairs had both siblings accepted or both rejected in the true population of origin, (2) how many had one sibling accepted into a reference population and the other sibling was rejected, and (3) how many sibling pairs had both offspring rejected or both accepted as belonging to any of the three reference populations considered rather than the true one (Supplementary Table S5).

For the 65 sibling pairs, the number of pairs rejected in all reference populations varied from one for the 122 SNPs to six for the 210 AIMs. The number of sibling pairs where one individual was accepted and one was rejected as belonging to any of the tested populations varied from seven (for the 46 indels) to 15 cases (for the 122 SNPs) (see Supplementary Figure S10). The 46 indels showed the highest sensitivity (percentage of sibling pairs that were not rejected in the true population), and the lowest sensitivity was obtained for the 210 AIMs (Supplementary Table S5 and Supplementary Figures S9, S10). Supplementary Table S5 presents the percentage of cases where individuals were accepted in their true population of origin and rejected in other reference populations, which indicates the specificity of each panel. In all cases, profiles were rejected as belonging to the NAM reference population. In two cases, one of the two siblings was also accepted in the AFR population. The highest proportion of ambiguities corresponded to profiles where individuals were both accepted in EUR and the Rio de Janeiro populations. The panel showing the lowest specificity was the 46 indels, and the 55 SNP panel was the one with the highest specificity (Supplementary Table S5). Taking together both sensitivity and specificity, the best results were obtained for the 55 SNPs, with 79.23% of acceptance in Rio de Janeiro and exclusion from other populations. The frequency of rejection of the true population plus ambiguous assignment was the highest for the 46 indels (44.62%) and varied from approximately 20 to 30% for the remaining panels.

The results obtained for the sibling pairs with different acceptance output in the true population (one accepted and one rejected), for at least one marker set, are described in Supplementary Figure S11. The highest agreement between siblings (both rejected or accepted) was obtained for the 46 indels. The number of sibling pairs with a different outcome was 13 for the 55 SNPs, 164 SNPs, and 210 AIMs, increasing to 15 for 122 SNPs. Except in one case (F87), the acceptance/rejection result varied among panels.

**FIGURE 14:**
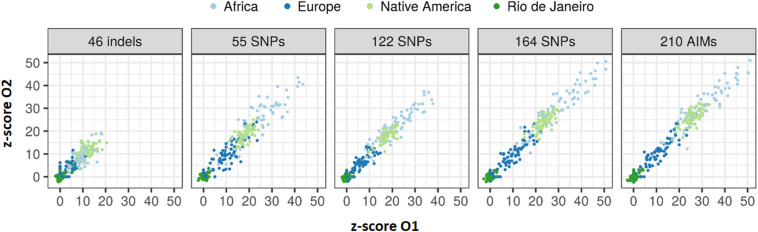
Values of z-score for the 65 sibling pairs when tested against Rio de Janeiro, AFR, EUR, and NAM population samples.

The z-scores calculated for the 65 sibling pairs considering in the four reference populations (Figure 14) showed that despite their low sensibility and specificity, larger panels resulted in higher rejection values when considering AFR, EUR, and NAM as possible populations of origin. A good agreement can also be seen in the z-scores between siblings.

## Discussion

### Genetic Profile of the Rio de Janeiro Population

Several studies have pointed to a high variation in the genetic background of Brazilian populations, that present different proportions of EUR, AFR, and NAM admixture. This characteristic is shared by most populations in South American countries (Salzano and Sans, 2014; Homburger et al., 2015; Chacón-Duque et al., 2018). The results from the analysis of 210 AIMs in the 214 unrelated individuals from Rio de Janeiro indicated that the population was predominantly European (54.0%) with admixture of African (38.5%) and Native American genetic heritage (7.5%), which was in accordance with the expectations based on previous studies on Brazilian populations (e.g., Pena et al., 2011; Manta et al., 2013; Salzano and Sans, 2014; Moura et al., 2015).

A comparison with another sample from Rio de Janeiro (Manta et al., 2013) showed differences in ancestry estimates. These differences can be explained by different sampling strategies in association with population stratification. Locus by locus analysis did not reveal statistically significant deviations to the HWE, except for one locus. Nevertheless, an overall excess of homozygotes was observed, particularly for loci showing large differences in allele frequency between the two main source populations (AFR and EUR). This excess of homozygosity is also supportive of population stratification in Rio de Janeiro.

In forensic genetics, it is important to consider the population stratification in the definition of allele frequency databases, and sub-structuring levels are also relevant for adjusting match probabilities (Curran et al., 2002; Buckleton et al., 2016; Hessab et al., 2018). In contrast to many North American populations, there are admixture gradients within populations in South America, which makes it difficult to define ethnic subgroups except for some Native and Afro-descendant communities that have maintained a certain degree of cultural identity and geographical isolation.

The 210 AIMs are essentially biallelic and a smaller number of individuals is usually necessary for accurate allele frequency estimations compared to multiallelic STRs. However, large sample sizes are required to detect HWE deviations and linkage disequilibrium that are more likely to occur in recently admixture and/or stratified populations (Kling et al., 2015).

No deviations from HWE have so far been reported for the commonly used STRs in admixed Brazilian populations (Rodrigues et al., 2007; de Assis Poiares et al., 2010; Alves et al., 2014; Hessab et al., 2015; Moyses et al., 2017). This may be attributed to the relatively small sample sizes since small deviations can only be detected in large samples. Furthermore, STRs selected for forensic identification have a high intrapopulation diversity and low intercontinental variability. Therefore, they are less efficient for the detection of HWE deviations in admixed populations than AIMs. In most forensic genetic publications, the authors employ Bonferroni adjustments whenever HWE *p*-values surpass the predefined significance level (usually 5%). However, no further consideration concerning the result itself or the sample size is usually made, which neglects the possibility of population stratification (Ye et al., 2020).

Lineage markers may also be useful to detect intrapopulation substructure since they present strong geographical differentiation. The presence of gametic associations between autosomal, mtDNA, and Y-chromosomal markers can be due to recent admixture and population stratification (Vullo et al., 2015). A study carried out in the Brazilian population of Rio de Janeiro showed a gametic association between autosomal AIMs and mtDNA haplogroups. This association between unlinked markers supports our hypotheses regarding the presence of population substructure in Rio de Janeiro (Simão et al., 2018). In summary, the results obtained in this study highlight the importance of having large sample sizes to investigate population substructure in admixed populations. Although statistically significant deviations to HWE could only be detected for a single marker when applying Bonferroni correction, the results indicated the need of studying a larger sample from Rio de Janeiro to investigate an overall excess of observed homozygosity.

### Ancestry Estimations in South American Admixed Populations

In the last few years, many studies have been published reporting new AIM sets to determine the proportion of intercontinental individual admixture and to infer BGA. Selected sets of different types of AIMs have been proposed based on their ability to determine population clustering patterns (Soundararajan et al., 2016; Kidd et al., 2017). In most cases, these panels were based on their ability to correctly assign the origin of individuals from African, Eastern Asian, European, Oceanian, and Native American populations. Less often, a higher resolution was pursued within one of these five groups (e.g., Li et al., 2016; Bulbul et al., 2018; Jung et al., 2019; Verdugo et al., 2020). Regardless of the ability of these sets to separate populations from different continents or geographic regions, the uncertainty associated with the estimates provided by these panels and their capacity to accurately report the different ancestral contributions in individuals of admixed populations has rarely been investigated.

This work aimed to compare the results of different groups of AIMs currently in use in the forensic field and their ability to determine the admixture proportions of a population, the profile of an individual’s ancestry, and the assignment of its population of origin in admixed populations from South America.

At the population level, all AIM sets reported similar population profiles in terms of the relative proportions of AFR, EUR, and NAM components in the seven admixed American populations. However, the absolute ancestry values were quite variable. Comparisons made for panels with different numbers of markers and different ability to differentiate the three main reference populations showed that the differences obtained were a function of these two variables. Depending on the profile of the population, it was observed that the performance of the studied AIM sets was related to the differentiation levels between reference populations as well as the equilibrium between these values. Therefore, obtaining reliable ancestry estimates in Admixed American populations not only depends on the selection of markers with high differentiation capacity but also on a balance of the differentiation values between the source populations (Galanter et al., 2012; Kidd et al., 2014; Phillips, 2015). The present study showed that the populations with the highest NAM ancestry were those, whose estimates had increased associated error. For these populations, this study also showed that more accurate estimates can be obtained when analyzing the 46 indels from Pereira et al. (2012) and the 164 SNPs of the Precision ID Ancestry panel together.

The discrepancies observed among panels at the individual level were higher than those at the population level. Particularly for the NAM component, the large differences observed in all populations regardless of the panel point to low accuracies of the estimates. These differences were also observed between the ancestries of parents vs. offspring, as well as between full siblings from the Rio de Janeiro population. The correlation and agreement between the ancestry estimates increased with the number of markers analyzed.

The high correlation and agreement between parents vs. offspring showed that this can be a good strategy for the evaluation of the performance of different panels. Although the admixture-enabled selection was described in the same Latin American populations that we studied from the 1000 genomes (Norris et al., 2020), this phenomenon was restricted to coding genes and not expected for the markers included in most of the sets selected for forensic use.

In forensic genetics, AIMs can be useful for BGA inference, as an investigative lead in the absence of a suspect (Phillips, 2015; Mogensen et al., 2020). To this end, it is, however, necessary that the relevant population is included in the investigated database. To evaluate this, Tvedebrink et al. (2018) derived a measure of agreement (z-score) that indicates whether a profile may come from a population that is represented within those being assessed. The results of the z-score analysis in 65 sibling pairs from Rio de Janeiro resulted in a large number of AIM profiles that were outliers in the true population. There was also a high number of ambiguous results, most of which were profiles that could belong to Rio de Janeiro and European populations. Moreover, increasing the number of AIMs did not increase the sensibility, although the specificity was higher. It is worth noting that no other South American populations were included, which would most certainly reduce the specificity even more. These results point out the complexity of BGA inference in highly admixed populations as those from South America and the large variation in the admixture proportions present in the population from Rio de Janeiro.

In a recent study, Pfaffelhuber et al. (2020) found high misclassification errors for the continental origin when Admixed American populations are included in the analysis of BGA. These authors concluded that, even for the AIM sets with the best performance in BGA inferences, when Admixed American populations were considered the misclassification was too large (30%) for forensic applications.

In summary, we illustrated the differences that can be expected when inferring ancestry or the populational origin of genetic profiles from South American admixed populations. Similar differences are expected to be present in other AIM sets with comparable characteristics in terms of the number of markers and genetic differentiation among source populations. Ancestry estimates are not only influenced by the number of markers included in the panel, but it is also essential to assess the level of differentiation that these markers provide among the reference populations. As seen in this work, there is a fine balance in the interplay of these factors.

The analysis of ancestry estimates at the population and individual levels helped to disclose what aspects to consider when selecting markers for an ancestry inference panel. Nevertheless, ancestry analyses will always present some degree of error when performing individual and population assignments. The focus should be to identify strategies for marker selection that minimize the error rate and increase the accuracy of the ancestry inference. Notwithstanding, the results obtained showed that even when the differences in estimates at the population level were minimized through the selection of a balanced group of markers or the use of the combined set, the errors at the individual level remained too high, demonstrating the need for a much higher number of markers for this purpose.

In the future, it would be interesting to perform investigations considering panels with higher resolution and also explore admixed populations with different number of source contributors to compare how the number of parental populations influences the ancestry results for different AIM panels.

Although it was not the scope in this work, an aspect to consider when inferring ancestry is the impact of the selection of appropriate reference populations. The admixture patterns in South America present differential contributions of several African and European populations from different regions along the continent. As an example, recent studies have attested that the presence of Northern Europeans is more restricted to the South, whereas Western European admixture events are more generalized (Montinaro et al., 2015; Gouveia et al., 2020).

The panels evaluated in this work have been designed to maximize differences between continents and are commonly used to ascertain main continental ancestry contributions. Indeed, previous studies reported absence of fine resolution within Sub-Saharan African, European, and East Asian groups (Al-Asfi et al., 2018; Lee et al., 2018; Nakanishi et al., 2018; Mogensen et al., 2020). Finer-scale admixture patterns within the South American continent have most recently been addressed with genome wide studies based on high density SNP data (Montinaro et al., 2015; Chacón-Duque et al., 2018; Ongaro et al., 2019; Gouveia et al., 2020). These studies have attested the complexity of the admixture dynamics of South America.

For the purposes of direct comparison of different datasets and other literature data, we have considered 100 Yorubans, 100 Central and British Europeans, and 47 Native Americans from several groups as references for all the populations studied. We used all available data for Native Americans and selected a random subset of 100 Africans and Europeans, to avoid large differences in the effective size between reference datasets. These individuals (and the reduced sample size of each reference group) are not necessarily the most appropriate references when looking particularly at the history of the Rio de Janeiro population. However, this work aimed to investigate how ancestry inferences fluctuate according to the number of loci used, the balance of the AIM panels, and the differentiation these AIMs provide. As such, the number and populations used for reference data will have minor impact on the conclusions of the study. Nevertheless, we should highlight that when assessing ancestry patterns for population and forensic genetic studies, it is important to consider the specific history of each population, and select a collection of reference individuals that is representative and better reflects those events.

## Data Availability Statement

The original contributions presented in the study are included in the article/Supplementary Material, further inquiries can be directed to the corresponding author.

## Ethics Statement

The studies involving human participants were reviewed and approved by the Comitê de Ética em Pesquisa, Universidade do Estado do Rio de Janeiro. The patients/participants provided their written informed consent to participate in this study.

## Author Contributions

VP and LG conceived and supervised the study, and wrote the first draft of the manuscript. RS and AA were responsible for samples collection, DNA extraction, and genotyping. VP, TT, and LG performed the statistical analysis of the data. CB and NM helped with data interpretation and manuscript drafting. All authors critically revised and approved the final manuscript.

## Conflict of Interest

The authors declare that the research was conducted in the absence of any commercial or financial relationships that could be construed as a potential conflict of interest.
