# Supplementary material for: Evaluation of the Precision of Ancestry Inferences in South American Admixed Populations
Source: Front Genet. 2020 Aug 21;11:966. doi: 10.3389/fgene.2020.00966 (PMC7472784; doi:10.3389/fgene.2020.00966)
Supplement: Supplementary file 1 [file Data_Sheet_1.zip › Data Sheet 1.PDF]

Supplementary Material

1 Supplementary Data

Supplementary Information 1.

AIM selection criteria

1. For each locus, we calculated the differential in allele frequencies between AFR-EUR, AFR-NAM, and EUR-NAM by dividing the highest value by the lowest one.

2. For each AIM panel, the cumulative value of differentiation for the three pairwise reference group comparisons was obtained by multiplying the values for each locus. The balance of the panel selection was confirmed by calculating pairwise F_{ST} genetic distances using the Arlequin software v3.5.2.2.

3. Three AIM panels were designed:

- The 46 panel B and 55 panel B were designed to include the same number of markers as 46 indels and 55 SNPs but selecting those markers that would result in more balanced pairwise FSTs among reference groups. Preference was given to the distance between EUR-NAM, which is the smallest in the original panels.

- The 40 AIMs panel was designed to contain markers that minimized the differences in pairwise FSTs between reference groups, by excluding markers from the 55 panel B.

**46 panel B**	MID128	MID196	MID1470	MID1802	MID2241	MID2538
	MID3626	rs10108270	rs10513300	rs11227699	rs12498138	rs1369093
	rs1426654	rs16891982	rs174570	rs1834619	rs1871534	rs1876482
	rs2024566	rs2042762	rs214678	rs2196051	rs2306040	rs2416791
	rs260690	rs2702414	rs2814778	rs2835370	rs3737576	rs3827760
	rs3907047	rs4717865	rs4746136	rs4833103	rs4951629	rs6451722
	rs647325	rs6541030	rs6548616	rs731257	rs7326934	rs734873
	rs735480	rs874299	rs9319336	rs9522149		
**55 panel B**	MID94	MID128	MID196	MID1470	MID1802	MID2005
	MID2241	MID2256	MID2538	MID3626	rs10108270	rs10513300
	rs11227699	rs12498138	rs12544346	rs1369093	rs1426654	rs16891982
	rs174570	rs1760921	rs1834619	rs1871534	rs1876482	rs2024566
	rs2030763	rs2042762	rs2125345	rs214678	rs2166624	rs2196051
	rs2306040	rs2330442	rs2416791	rs260690	rs2702414	rs2814778
	rs2835370	rs3737576	rs3827760	rs3907047	rs4717865	rs4746136
	rs4833103	rs4951629	rs6451722	rs647325	rs6541030	rs6548616
	rs731257	rs7326934	rs734873	rs735480	rs874299	rs9319336
	rs9522149					
**40 AIMs**	MID128	MID196	MID1470	MID1802	MID2241	MID2538
	MID3626	rs10108270	rs11227699	rs12498138	rs1369093	rs1426654
	rs16891982	rs174570	rs1834619	rs1871534	rs1876482	rs2024566
	rs2030763	rs214678	rs2196051	rs2330442	rs2416791	rs260690
	rs2702414	rs2835370	rs3737576	rs3827760	rs4833103	rs4951629
	rs6451722	rs647325	rs6541030	rs6548616	rs731257	rs7326934
	rs735480	rs874299	rs9319336	rs9522149		

2 Supplementary Figures and Tables

2.1 Supplementary Figures

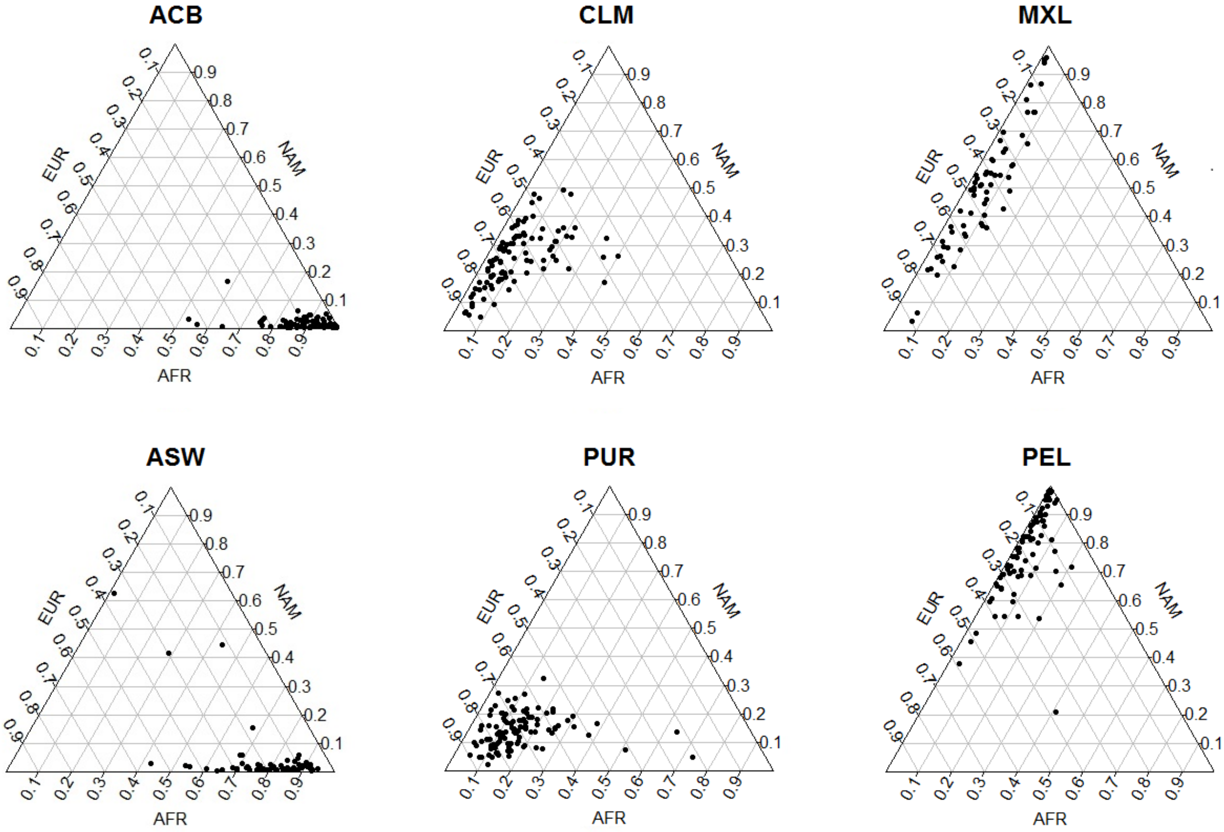

Supplementary Figure S1. Triangular plot of the q-matrices generated in STRUCTURE and combined in CLUMPP, considering the distribution of the three ancestry components in each individual based on 210 AIMs, for the six population samples: African Caribbean in Barbados (ACB); Americans of African ancestry in Southwest USA (ASW); Colombians from Medellin, Colombia (CLM); Mexican Ancestry from Los Angeles, USA (MXL); Peruvians from Lima, Peru (PEL) and, Puerto Ricans from Puerto Rico (PUR).

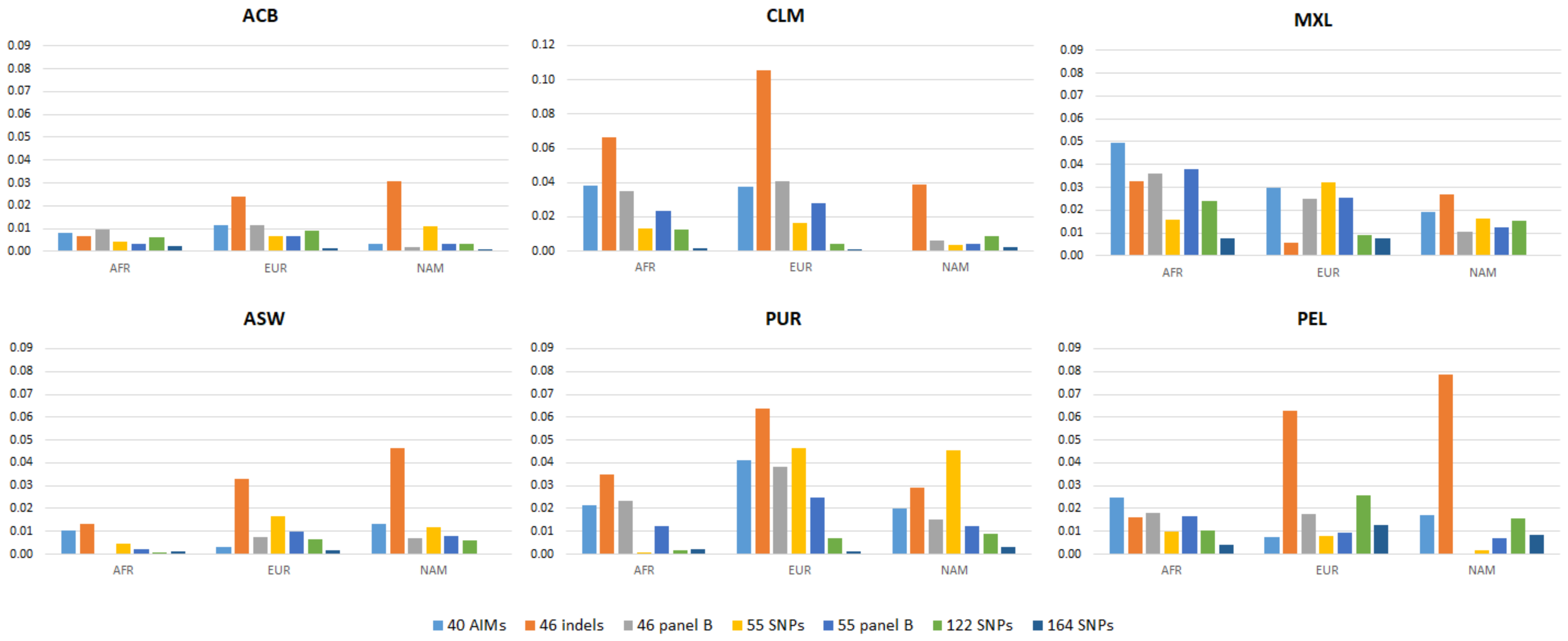

Supplementary Figure S2. Absolute values of the differences between the average ancestries reported for each panel compared to the 210 AIMs, for the six population samples: African Caribbean in Barbados (ACB); Americans of African ancestry in Southwest USA (ASW); Colombians from Medellin, Colombia (CLM); Mexican Ancestry from Los Angeles, USA (MXL); Peruvians from Lima, Peru (PEL) and, Puerto Ricans from Puerto Rico (PUR).

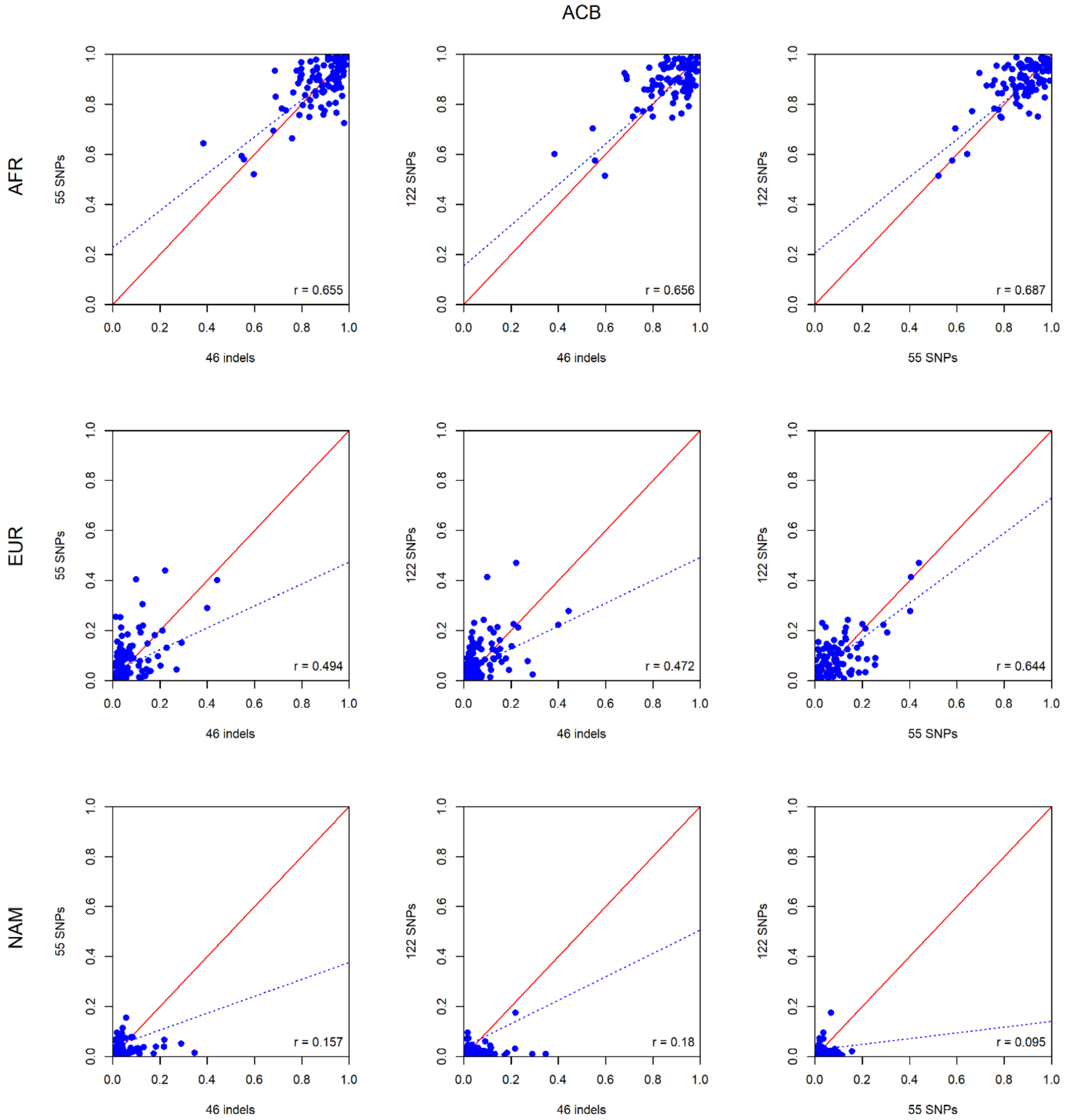

Supplementary Figure S3. Pairwise comparisons between ancestry estimates provided by 46 indels, 55 SNPs and 122 SNPs in individuals from the African Caribbean population in Barbados (ACB). The respective r-values are indicated in the figures, and the tendency line is represented as a blue dashed line. The red solid line indicates the perfect agreement between two AIM panels.

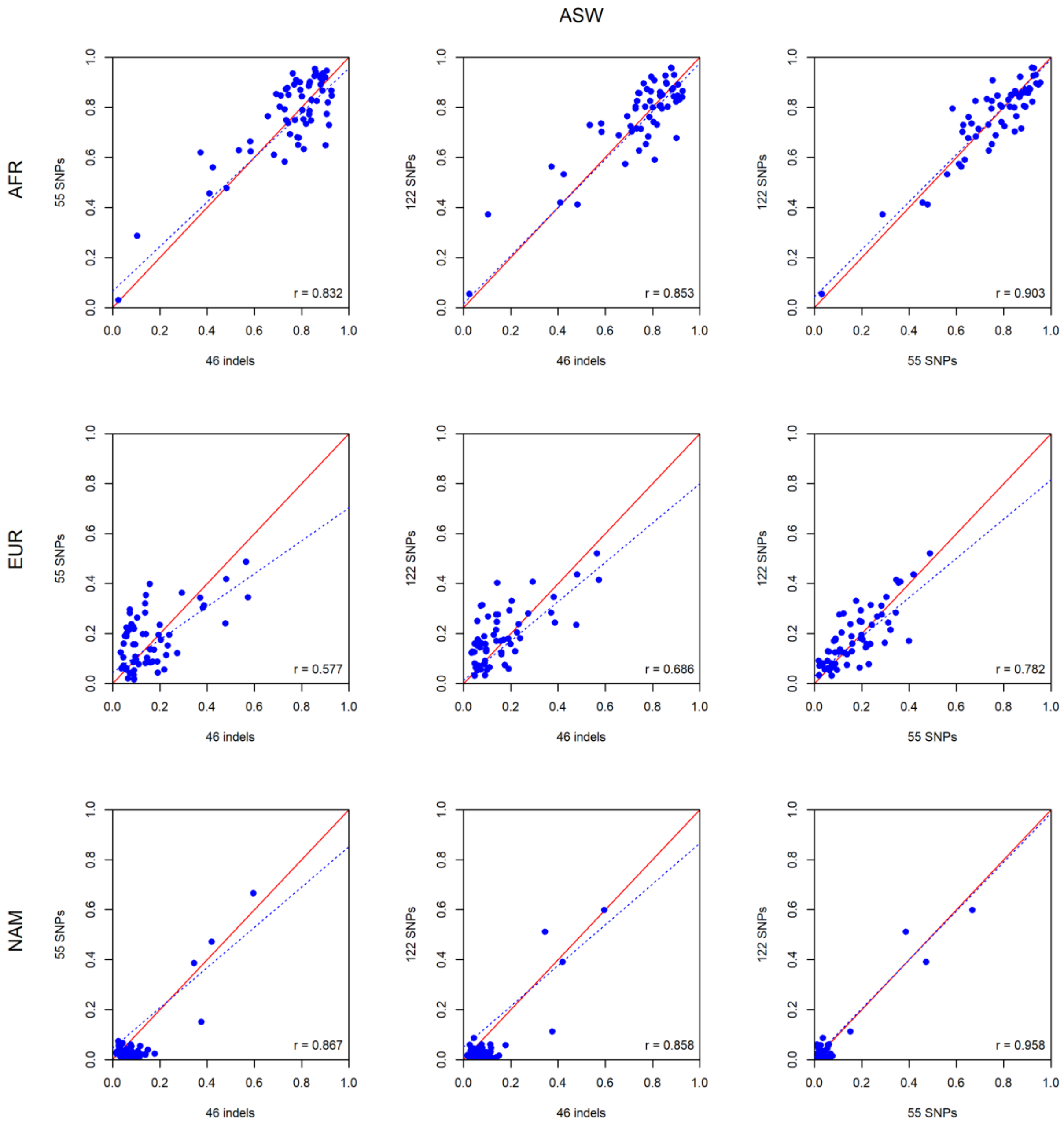

Supplementary Figure S4. Pairwise comparisons between ancestry estimates provided by 46 indels, 55 SNPs, and 122 SNPs in Americans of African ancestry in Southwest USA (ASW). The respective r-values are indicated in the figures, and the tendency line is represented as a blue dashed line. The red solid line indicates the perfect agreement between two AIM panels.

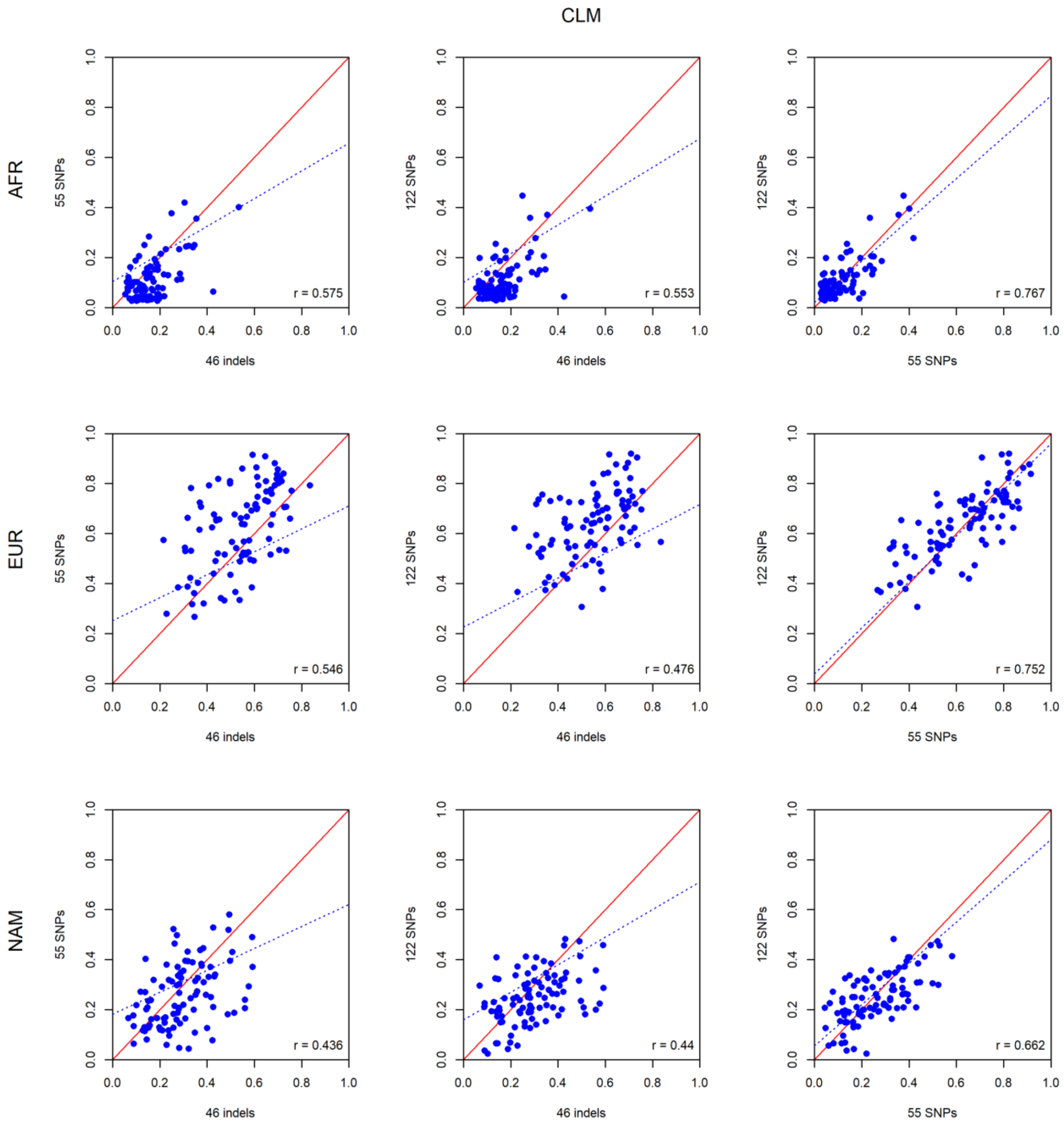

Supplementary Figure S5. Pairwise comparisons between ancestry estimates provided by 46 indels, 55 SNPs, and 122 SNPs in individuals from Medellin, Colombia (CLM). The respective r-values are indicated in the figures, and the tendency line is represented as a blue dashed line. The red solid line indicates the perfect agreement between two AIM panels.

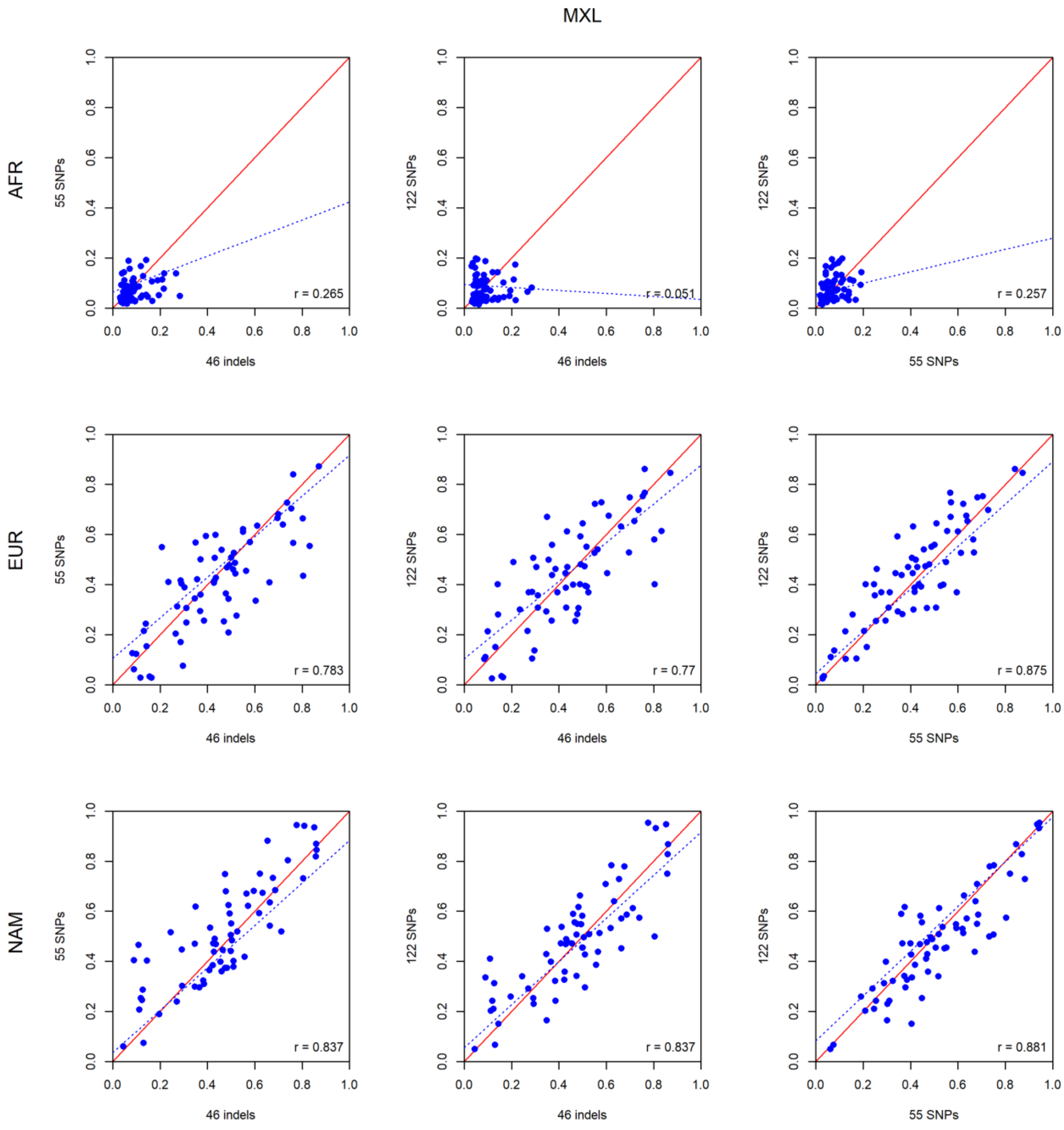

Supplementary Figure S6. Pairwise comparisons between ancestry estimates provided by 46 indels, 55 SNPs, and 122 SNPs in Mexican Ancestry individuals from Los Angeles, USA (MXL). The respective r-values are indicated in the figures, and the tendency line is represented as a blue dashed line. The red solid line indicates the perfect agreement between two AIM panels.

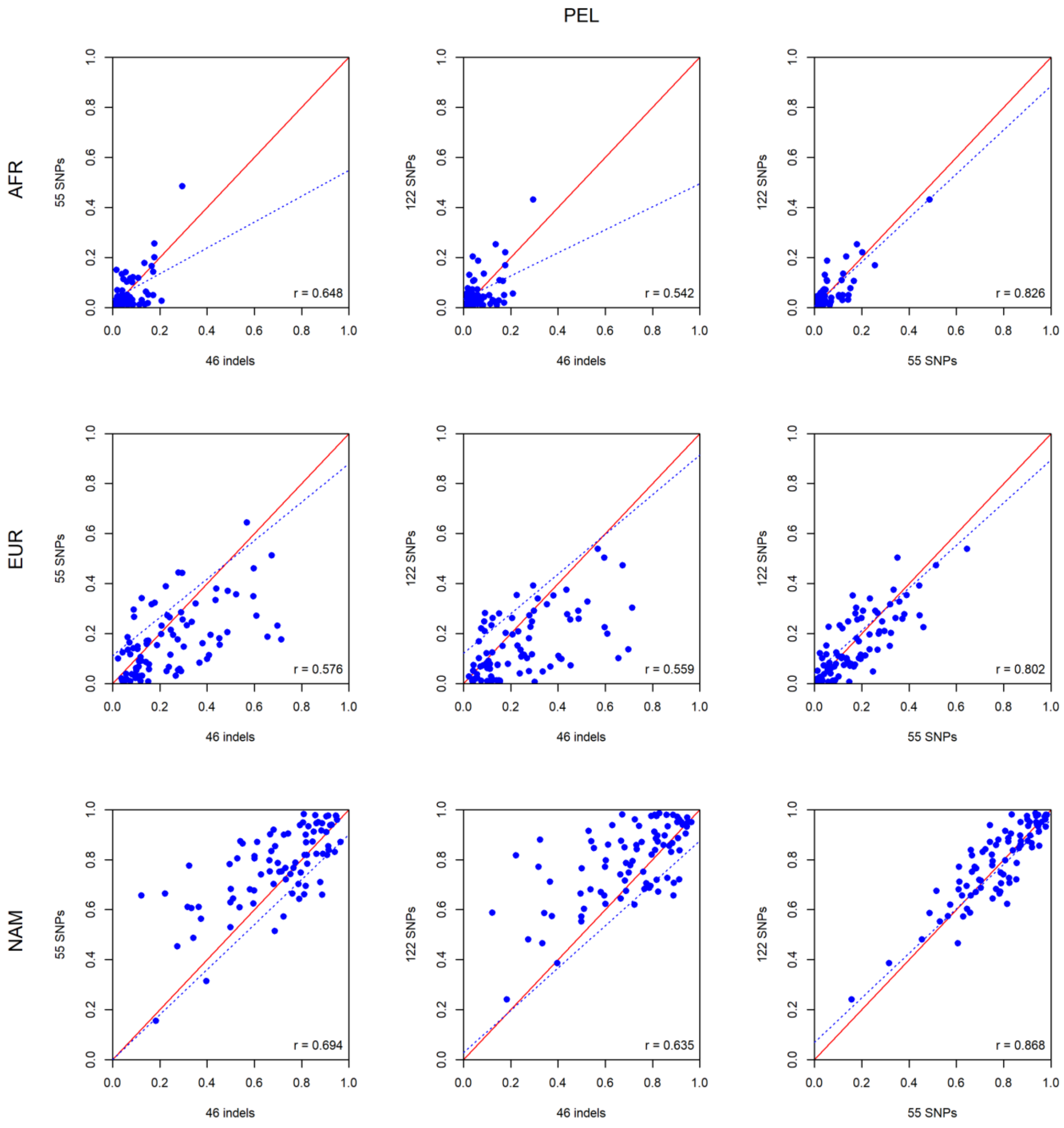

Supplementary Figure S7. Pairwise comparisons between ancestry estimates provided by 46 indels, 55 SNPs, and 122 SNPs in Peruvians from Lima, Peru (PEL). The respective r-values are indicated in the figures, and the tendency line is represented as a blue dashed line. The red solid line indicates the perfect agreement between two AIM panels.

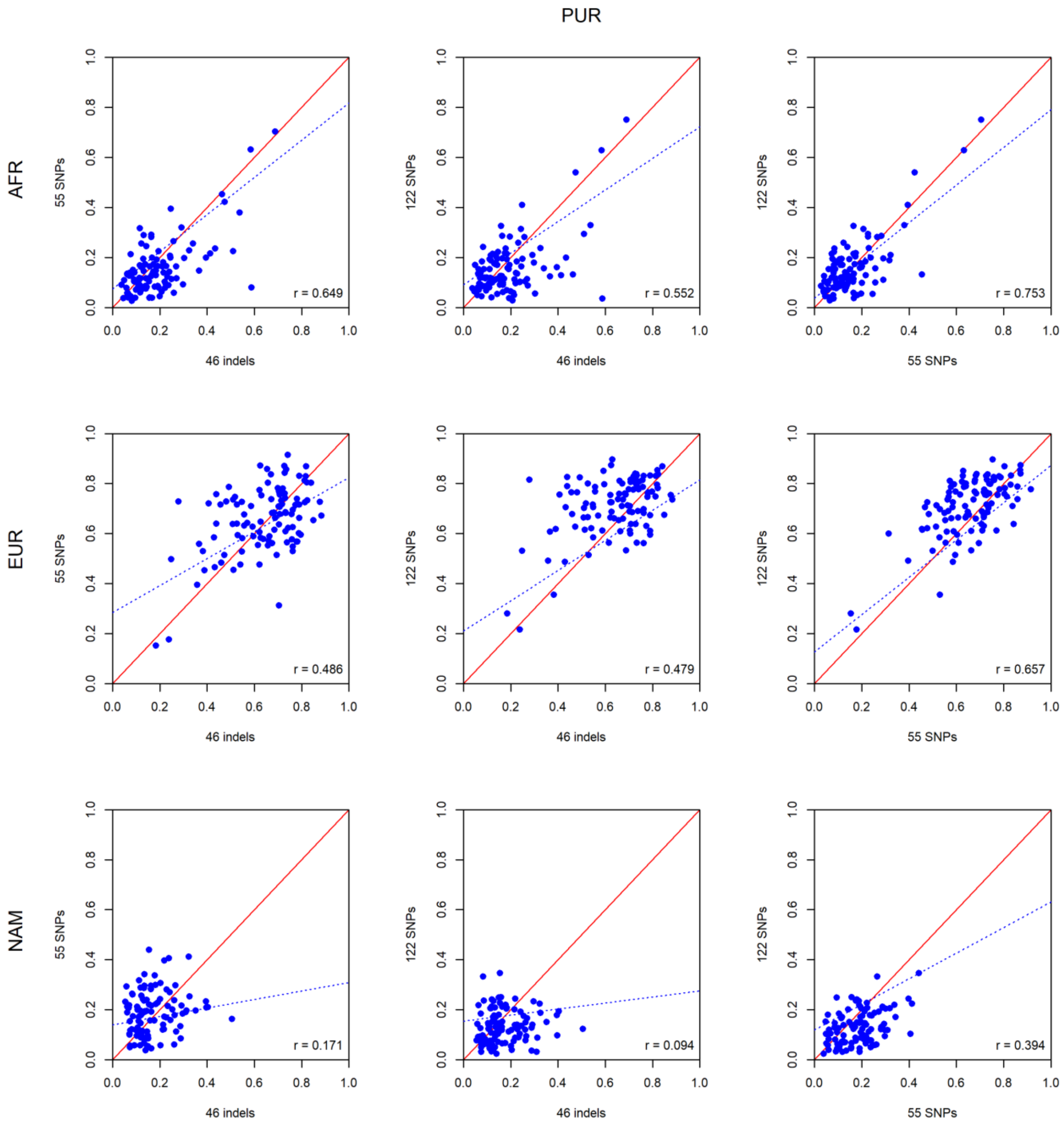

Supplementary Figure S8. Pairwise comparisons between ancestry estimates provided by 46 indels, 55 SNPs, and 122 SNPs in unrelated individuals from Puerto Rico (PUR). The respective r-values are indicated in the figures, and the tendency line is represented as a blue dashed line. The red solid line indicates the perfect agreement between two AIM panels.

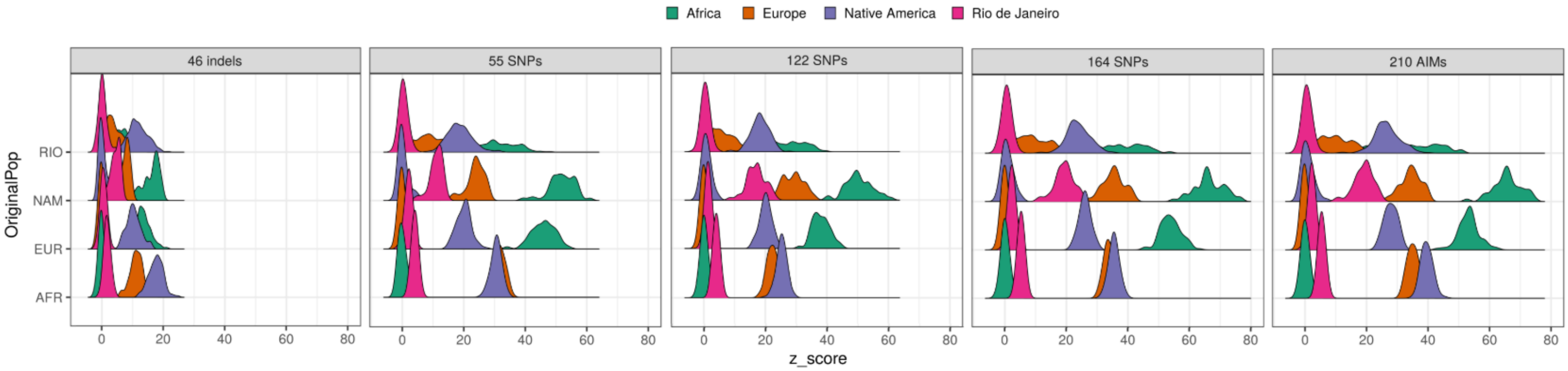

Supplementary Figure S9. Distributions of the z-scores obtained for 100 AFR, 100 EUR, 47 NAM and 214 Rio de Janeiro individuals tested using 5 AIM-sets. Each line shows the smooth density histogram of the observed z-scores for each reference populations indicated to the left.

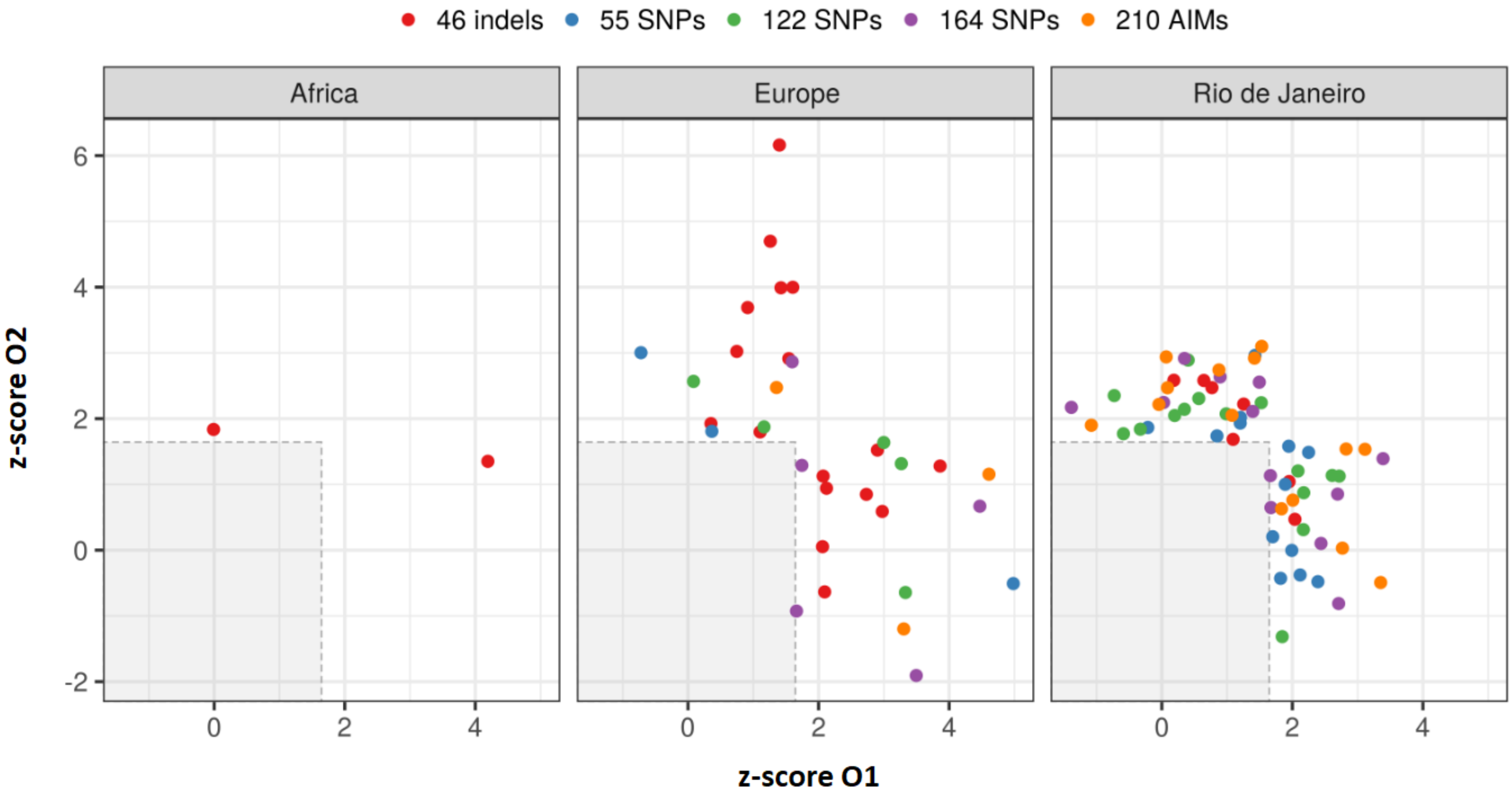

Supplementary Figure S10. Representation of the z-scores obtained in sibling pairs (O1 and O2) with different acceptance output in the true population (one accepted and one rejected). Note that no profile was accepted in the Native American population. The light grey lines at z = 1.64 shows the 95%-quantile for the standard normal distribution.

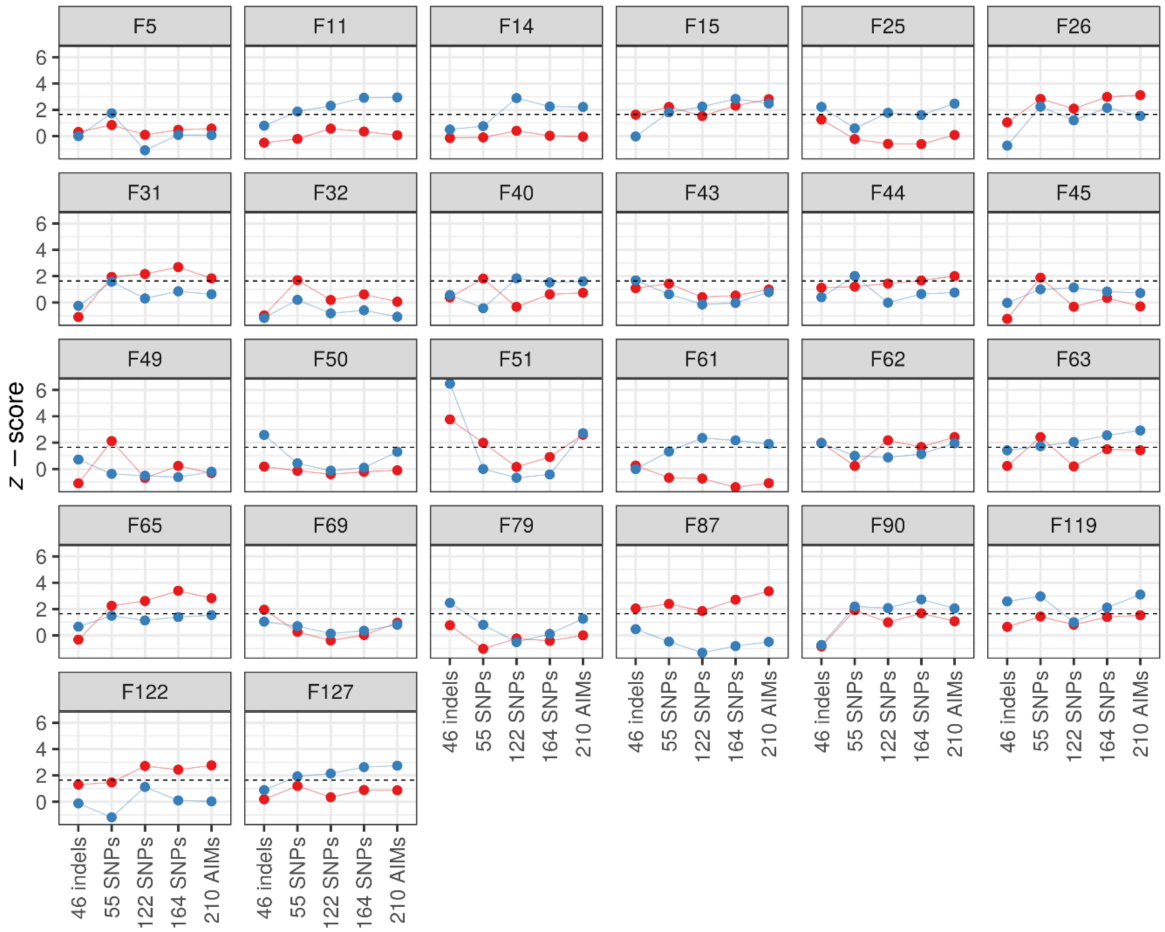

Supplementary Figure S11. Values of z-score test for the 26 sibling pairs where siblings had different acceptance status (one accepted and one rejected) when tested against Rio de Janeiro population sample. The blue and red dots correspond to the z-scores of the siblings in the five panels. The dotted lines at z = 1.64 show the 95%-quantile for the standard normal distribution. For values above this line, Rio de Janeiro is rejected as a plausible population of origin of the profile.

2.2 Supplementary Tables

Supplementary Table S1. Allele frequency information for the 210 ancestry markers analyzed, in the three reference populations and the sample of unrelated individuals from Rio de Janeiro.

Marker	Dataset	Allele	AFR (N=100)	EUR (N=100)	NAM (N=47)	RIO UNRELATED (N=214)
**MID1470 (rs144826367)**	46plex	**1**	0.22	0.66	0.01	0.40
**MID777 (rs5815316)**	46plex	**1**	0.43	0.36	0.86	0.35
**MID196 (rs16635)**	46plex	**1**	0.54	0.47	0.05	0.48
**MID881 (rs3072025)**	46plex	**1**	0.39	0.93	1.00	0.69
**MID3122 (rs35451359)**	46plex	**1**	0.59	0.98	1.00	0.80
**MID548 (rs140837)**	46plex	**1**	0.43	0.23	0.14	0.30
**MID659 (rs1160893)**	46plex	**1**	0.37	0.05	0.28	0.18
**MID2011 (rs2308203)**	46plex	**1**	0.20	0.78	0.93	0.55
**MID2929 (rs33974167)**	46plex	**1**	0.77	0.75	0.98	0.70
**MID593 (rs1160852)**	46plex	**1**	0.71	0.01	0.01	0.32
**MID798 (rs33966939)**	46plex	**1**	0.16	0.66	0.12	0.39
**MID1193 (rs2067280)**	46plex	**1**	0.15	0.14	0.16	0.17
**MID1871 (rs2308067)**	46plex	**1**	0.03	0.36	0.30	0.18
**MID17 (rs4183)**	46plex	**1**	0.66	0.28	0.91	0.53
**MID2538 (rs3054057)**	46plex	**1**	0.00	0.45	0.04	0.29
**MID1644 (rs2307840)**	46plex	**1**	0.21	0.95	0.15	0.65
**MID3854 (rs3839445)**	46plex	**1**	0.69	0.02	0.01	0.28
**MID2275 (rs3033053)**	46plex	**1**	0.43	0.09	0.59	0.22
**MID94 (rs16384)**	46plex	**1**	0.00	0.19	0.66	0.18
**MID3072 (rs67532422)**	46plex	**1**	0.07	0.96	0.98	0.64
**MID772 (rs1610859)**	46plex	**1**	0.96	0.97	0.77	0.88
**MID2313 (rs3045215)**	46plex	**1**	0.14	0.27	0.71	0.29
**MID397 (rs25621)**	46plex	**1**	0.82	0.81	0.33	0.69
**MID1636 (rs2307832)**	46plex	**1**	0.24	0.75	1.00	0.58
**MID51 (rs16343)**	46plex	**1**	0.09	0.60	0.88	0.40
**MID2431 (rs3031979)**	46plex	**1**	0.12	0.09	0.26	0.16
**MID2264 (rs34122827)**	46plex	**1**	0.28	0.52	0.14	0.43
		**2**	0.73	0.48	0.86	0.54
		**2-T**	0.00	0.00	0.00	0.04
**MID2256 (rs133052)**	46plex	**1**	0.00	0.25	0.85	0.21
**MID128 (rs35721831)**	46plex	**1**	0.00	0.41	0.03	0.25
**MID15 (rs4181)**	46plex	**1**	0.25	0.44	0.84	0.38

**MID2241 (rs3030826)**	46plex	**1**	0.82	0.30	0.06	0.49
**MID419 (rs140708)**	46plex	**1**	0.98	0.78	0.50	0.80
**MID943 (rs1611026)**	46plex	**1**	0.32	0.85	0.40	0.58
**MID159 (rs16438)**	46plex	**1**	0.75	0.55	0.76	0.64
		**2**	0.26	0.45	0.24	0.35
		**2-T**	0.00	0.00	0.00	0.00
**MID2005 (rs10651200)**	46plex	**1**	0.03	0.70	0.29	0.40
**MID250 (rs16687)**	46plex	**1**	0.78	0.71	0.39	0.70
**MID1802 (rs2307998)**	46plex	**1**	0.51	0.02	0.00	0.19
**MID1607 (rs2307803)**	46plex	**1**	0.41	0.20	0.57	0.23
**MID1734 (rs2307930)**	46plex	**1**	0.91	0.86	0.36	0.76
**MID406 (rs25630)**	46plex	**1**	0.04	0.80	0.70	0.46
**MID1386 (rs71798723)**	46plex	**1**	0.15	0.26	0.77	0.19
**MID1726 (rs2307922)**	46plex	**1**	0.28	0.74	0.76	0.47
**MID3626 (*)**	46plex	**1**	0.16	0.74	0.03	0.46
**MID360 (rs25584)**	46plex	**1**	0.60	0.82	0.50	0.69
		**2**	0.39	0.18	0.50	0.28
		**2-T**	0.02	0.00	0.00	0.03
**MID1603 (rs2307799)**	46 plex	**1**	0.09	0.35	0.81	0.33
**MID2719 (rs3830845)**	46plex	**1**	0.32	0.39	0.47	0.42
**rs10007810**	122 AIMs	**A**	1.00	0.23	0.28	0.51
**rs10108270**	122 AIMs	**A**	0.95	0.28	0.03	0.53
**rs10236187**	122 AIMs	**A**	0.42	0.96	0.36	0.71
**rs1040045**	122 AIMs	**A**	0.14	0.73	0.99	0.53
**rs1040404**	122 AIMs	**A**	0.92	0.26	0.79	0.61
**rs10496971**	122 AIMs	**G**	0.05	0.08	0.48	0.10
**rs10497191**	55 AIMs	**C**	0.03	0.86	0.91	0.55
**rs10511828**	122 AIMs	**C**	0.10	0.09	0.67	0.13
**rs10512572**	122 AIMs	**A**	0.24	0.14	0.69	0.22
**rs10513300**	122 AIMs	**C**	0.00	0.06	0.67	0.08
**rs1079597**	55 AIMs	**C**	0.81	0.87	0.30	0.82
**rs10839880**	122 AIMs	**C**	0.93	0.35	0.32	0.57
**rs11227699**	122 AIMs	**A**	0.65	0.05	0.55	0.26
**rs11652805**	55 AIMs; 122 AIMs	**C**	0.98	0.12	0.10	0.50
**rs12130799**	122 AIMs	**A**	1.00	0.90	0.55	0.94
**rs1229984**	55 AIMs	**C**	1.00	0.99	0.98	0.95
**rs12439433**	55 AIMs; 122 AIMs	**A**	1.00	0.96	0.55	0.96

**rs12498138**	55 AIMs	**A**	0.00	0.05	0.88	0.09
**rs12544346**	122 AIMs	**A**	0.00	0.60	0.45	0.36
**rs12629908**	122 AIMs	**A**	0.08	0.09	0.79	0.14
**rs12657828**	122 AIMs	**A**	0.76	0.86	0.26	0.77
**rs12913832**	55 AIMs	**A**	1.00	0.23	0.85	0.77
**rs1296819**	122 AIMs	**A**	0.84	0.26	0.24	0.45
**rs1325502**	122 AIMs	**A**	0.79	0.17	0.07	0.41
**rs13400937**	122 AIMs	**G**	0.93	0.21	0.27	0.55
**rs1369093**	122 AIMs	**C**	0.39	0.05	0.73	0.20
**rs1407434**	122 AIMs	**A**	0.24	0.11	0.85	0.20
**rs1426654**	55 AIMs	**A**	0.02	1.00	0.04	0.54
**rs1462906**	55 AIMs	**C**	0.20	0.98	1.00	0.69
**rs1471939**	122 AIMs	**C**	0.35	0.16	0.77	0.31
**rs1500127**	122 AIMs	**C**	0.47	0.97	0.45	0.75
**rs1503767**	122 AIMs	**G**	0.80	0.13	0.48	0.38
**rs1513056**	122 AIMs	**A**	0.37	0.16	0.86	0.30
**rs1513181**	122 AIMs	**A**	0.18	0.14	0.90	0.22
**rs1569175**	122 AIMs	**C**	0.71	0.95	0.27	0.87
**rs1572018**	55 AIMs	**C**	0.02	0.83	0.96	0.51
**rs16891982**	55 AIMs	**C**	1.00	0.02	0.93	0.58
**rs174570**	55 AIMs	**C**	0.99	0.84	0.05	0.84
**rs1760921**	122 AIMs	**C**	0.75	0.05	0.11	0.31
**rs17642714**	55 AIMs	**A**	1.00	0.71	0.88	0.83
**rs1800414**	55 AIMs	**T**	1.00	1.00	1.00	1.00
**rs1834619**	55 AIMs	**A**	0.01	0.03	0.97	0.07
**rs1837606**	122 AIMs	**C**	0.71	0.30	0.79	0.50
**rs1871428**	122 AIMs	**A**	0.99	0.33	0.80	0.67
**rs1871534**	55 AIMs	**C**	0.97	0.00	0.03	0.34
**rs1876482**	55 AIMs	**A**	0.00	0.02	0.41	0.05
**rs1879488**	122 AIMs	**A**	0.18	0.08	0.69	0.18
**rs192655**	55 AIMs; 122 AIMs	**A**	0.55	0.93	0.37	0.73
**rs1950993**	122 AIMs	**G**	0.03	0.59	0.27	0.37
**rs2001907**	122 AIMs	**C**	0.99	0.94	0.45	0.90
**rs200354**	55 AIMs; 122 AIMs	**G**	0.29	0.86	0.11	0.54
**rs2024566**	55 AIMs	**A**	0.88	0.60	0.02	0.73
**rs2030763**	122 AIMs	**A**	0.02	0.19	0.74	0.14
**rs2033111**	122 AIMs	**A**	0.48	0.92	0.41	0.70
**rs2042762**	55 AIMs	**C**	0.00	0.04	0.55	0.06

**rs2070586**	122 AIMs	**A**	0.42	0.16	0.71	0.30
**rs2073821**	122 AIMs	**C**	1.00	0.92	0.40	0.91
**rs2125345**	122 AIMs	**C**	0.99	0.29	0.09	0.56
**rs214678**	122 AIMs	**C**	0.06	0.11	0.62	0.12
**rs2166624**	55 AIMs	**A**	0.00	0.39	0.99	0.26
**rs2196051**	55 AIMs	**A**	0.00	0.69	0.02	0.44
**rs2238151**	55 AIMs	**C**	1.00	0.35	0.55	0.59
**rs2306040**	122 AIMs	**C**	0.06	0.06	0.56	0.08
**rs2330442**	122 AIMs	**A**	0.02	0.60	0.18	0.38
**rs2357442**	122 AIMs	**A**	0.78	0.85	0.28	0.85
**rs2416791**	122 AIMs	**A**	0.97	0.09	0.51	0.44
**rs2504853**	122 AIMs	**C**	0.99	0.33	0.53	0.56
**rs2532060**	122 AIMs	**C**	0.97	0.41	0.61	0.67
**rs2593595**	55 AIMs	**A**	0.02	0.77	0.83	0.46
**rs260690**	55 AIMs; 122 AIMs	**A**	0.31	0.93	0.09	0.64
**rs2627037**	122 AIMs	**A**	0.48	0.11	0.64	0.24
**rs2702414**	122 AIMs	**A**	0.05	0.07	0.74	0.12
**rs2814778**	55 AIMs	**C**	1.00	0.00	0.03	0.38
**rs2835370**	122 AIMs	**C**	0.49	0.05	0.63	0.22
**rs2899826**	122 AIMs	**A**	0.62	0.92	0.23	0.71
**rs2946788**	122 AIMs	**G**	0.89	0.23	0.24	0.47
**rs2966849**	122 AIMs	**A**	0.31	0.12	0.79	0.25
**rs2986742**	122 AIMs	**C**	0.81	0.10	0.07	0.43
**rs310644**	55 AIMs	**C**	0.95	0.12	0.05	0.27
**rs3118378**	122 AIMs	**A**	0.54	0.66	0.08	0.62
**rs316598**	122 AIMs	**C**	1.00	0.24	0.30	0.61
**rs316873**	122 AIMs	**C**	0.92	0.87	0.37	0.89
**rs32314**	122 AIMs	**C**	0.32	0.28	0.97	0.30
**rs37369**	122 AIMs	**C**	0.30	0.91	0.30	0.65
**rs3737576**	55 AIMs; 122 AIMs	**C**	0.02	0.05	0.74	0.09
**rs3745099**	122 AIMs	**A**	0.10	0.88	0.65	0.59
**rs3784230**	122 AIMs	**A**	0.01	0.58	0.60	0.34
**rs3793451**	122 AIMs	**C**	0.98	0.96	0.64	0.90
**rs3793791**	122 AIMs	**C**	0.30	0.11	0.69	0.19
**rs3811801**	55 AIMs	**G**	1.00	1.00	1.00	1.00
**rs3814134**	55 AIMs	**A**	0.07	0.99	0.96	0.66
**rs3823159**	55 AIMs	**A**	0.10	1.00	0.62	0.63

**rs3827760**	55 AIMs	**A**	1.00	1.00	0.09	0.94
**rs385194**	122 AIMs	**A**	0.99	0.32	0.85	0.58
**rs3907047**	122 AIMs	**C**	0.00	0.07	0.66	0.06
**rs3916235**	55 AIMs	**C**	0.09	0.96	0.98	0.63
**rs3943253**	122 AIMs	**A**	0.67	0.93	0.20	0.80
**rs4411548**	55 AIMs	**C**	0.81	0.77	0.46	0.80
**rs4458655**	122 AIMs	**C**	0.45	0.14	0.77	0.27
**rs4463276**	122 AIMs	**A**	0.91	0.21	0.97	0.54
**rs4471745**	55 AIMs	**A**	0.09	0.07	0.01	0.05
**rs459920**	55 AIMs	**C**	0.15	0.43	0.11	0.36
**rs4666200**	122 AIMs	**A**	0.11	0.75	0.90	0.51
**rs4670767**	122 AIMs	**G**	0.99	0.91	0.55	0.90
**rs4717865**	122 AIMs	**A**	0.00	0.11	0.52	0.07
**rs4746136**	122 AIMs	**A**	0.00	0.19	0.82	0.10
**rs4781011**	122 AIMs	**G**	0.40	0.76	0.15	0.59
**rs4798812**	122 AIMs	**A**	0.24	0.28	0.71	0.28
**rs4821004**	122 AIMs	**C**	0.07	0.60	0.65	0.43
**rs4833103**	55 AIMs	**A**	0.00	0.59	0.02	0.20
**rs4880436**	122 AIMs	**C**	1.00	0.93	0.48	0.93
**rs4891825**	55 AIMs; 122 AIMs	**A**	0.04	0.88	0.88	0.59
**rs4908343**	122 AIMs	**A**	0.03	0.80	0.95	0.47
**rs4918664**	55 AIMs	**A**	1.00	0.90	0.11	0.92
**rs4918842**	122 AIMs	**C**	0.10	0.13	0.96	0.22
**rs4951629**	122 AIMs	**C**	0.15	0.05	0.55	0.17
**rs4955316**	122 AIMs	**G**	0.21	0.12	0.67	0.14
**rs4984913**	122 AIMs	**A**	0.09	0.70	0.27	0.49
**rs5768007**	122 AIMs	**C**	1.00	0.90	0.14	0.87
**rs6104567**	122 AIMs	**G**	0.09	0.29	0.88	0.24
**rs6422347**	122 AIMs	**C**	0.90	0.09	0.05	0.41
**rs6451722**	122 AIMs	**A**	0.92	0.21	0.03	0.47
**rs6464211**	122 AIMs	**C**	0.18	0.86	0.62	0.59
**rs647325**	122 AIMs	**A**	0.37	0.83	0.06	0.60
**rs6541030**	122 AIMs	**A**	0.27	0.04	0.37	0.18
**rs6548616**	122 AIMs	**C**	0.96	0.27	0.05	0.54
**rs6556352**	122 AIMs	**C**	0.01	0.67	0.35	0.44
**rs671**	55 AIMs	**G**	1.00	1.00	1.00	1.00
**rs6754311**	55 AIMs	**C**	1.00	0.25	0.98	0.75
**rs6990312**	55 AIMs	**G**	0.27	0.83	0.63	0.61

**rs705308**	122 AIMs	**A**	1.00	0.50	0.98	0.68
**rs7226659**	55 AIMs	**G**	0.97	0.96	0.59	0.93
**rs7238445**	122 AIMs	**A**	0.91	0.23	0.33	0.47
**rs7251928**	55 AIMs	**A**	0.08	0.74	1.00	0.52
**rs731257**	122 AIMs	**A**	0.02	0.15	0.90	0.14
**rs7326934**	55 AIMs	**C**	0.86	0.02	0.12	0.32
**rs734873**	122 AIMs	**A**	0.01	0.15	0.65	0.10
**rs735480**	55 AIMs	**C**	0.99	0.09	0.57	0.51
**rs7421394**	122 AIMs	**A**	0.03	0.70	0.43	0.43
**rs7554936**	55 AIMs; 122 AIMs	**C**	1.00	0.39	0.14	0.58
**rs7657799**	55 AIMs; 122 AIMs	**G**	0.84	0.04	0.02	0.32
**rs7722456**	55 AIMs	**C**	0.33	0.21	0.02	0.27
**rs772262**	122 AIMs	**A**	0.87	0.10	0.69	0.39
**rs7745461**	122 AIMs	**A**	0.38	0.12	0.56	0.26
**rs7803075**	122 AIMs	**A**	1.00	0.29	0.84	0.60
**rs7844723**	122 AIMs	**C**	1.00	0.42	0.81	0.65
**rs798443**	55 AIMs; 122 AIMs	**A**	0.03	0.82	0.80	0.53
**rs7997709**	55 AIMs; 122 AIMs	**C**	0.19	0.10	0.93	0.19
**rs8021730**	122 AIMs	**G**	0.21	0.84	0.54	0.55
**rs8035124**	122 AIMs	**A**	0.23	0.84	0.15	0.64
**rs8113143**	122 AIMs	**A**	1.00	0.34	0.81	0.59
**rs818386**	122 AIMs	**C**	1.00	0.81	0.38	0.89
**rs870347**	55 AIMs; 122 AIMs	**A**	0.96	0.92	0.27	0.90
**rs874299**	122 AIMs	**C**	0.04	0.74	0.13	0.45
**rs881728**	122 AIMs	**A**	0.12	0.12	0.60	0.14
**rs917115**	55 AIMs	**C**	0.81	0.20	0.85	0.46
**rs9291090**	122 AIMs	**A**	0.97	0.96	0.51	0.95
**rs9319336**	122 AIMs	**C**	0.15	0.07	0.71	0.14
**rs946918**	122 AIMs	**G**	0.85	0.80	0.22	0.76
**rs948028**	122 AIMs	**A**	0.22	0.87	0.74	0.61
**rs9522149**	55 AIMs; 122 AIMs	**C**	0.03	0.76	0.04	0.46
**rs9530435**	122 AIMs	**C**	0.11	0.84	0.96	0.56
**rs9809104**	122 AIMs	**C**	0.92	0.21	0.32	0.48
**rs9845457**	122 AIMs	**A**	0.02	0.63	0.90	0.40

* rs11267926 was deleted from dbSNP on Oct 17, 2013 due to mapping or clustering errors. No current new rs number is available.

Supplementary Table S2. Differences between expected and observed heterozygosity values for the 210 AIMs in the Rio de Janeiro sample. AIMs are presented in descending order of allele frequency differences between AFR and EUR (delta AFR-EUR, absolute value).

RIO DE JANEIRO UNRELATED INDIVIDUALS

MARKER	DELTA AFR-EUR	EXP HET	OBS HET	EXP-OBS
**rs2814778**	0.99	0.47381	0.42056	0.05325
**rs1426654**	0.98	0.49763	0.39252	0.10511
**rs16891982**	0.98	0.48686	0.40845	0.07841
**rs1871534**	0.97	0.44907	0.38785	0.06122
**rs3814134**	0.92	0.45205	0.43458	0.01747
**rs3823159**	0.91	0.46562	0.42523	0.04039
**rs735480**	0.91	0.5009	0.44393	0.05697
**MID3072**	0.89	0.46177	0.40187	0.0599
**rs2416791**	0.88	0.49487	0.42991	0.06496
**rs3916235**	0.88	0.46995	0.3986	0.07135
**rs11652805**	0.87	0.50116	0.46262	0.03854
**rs4891825**	0.84	0.48619	0.43458	0.05161
**rs7326934**	0.84	0.43961	0.38785	0.05176
**rs10497191**	0.83	0.49587	0.47664	0.01923
**rs310644**	0.83	0.39478	0.36111	0.03367
**rs1572018**	0.82	0.50079	0.41748	0.08331
**rs6422347**	0.81	0.48366	0.42991	0.05375
**rs7657799**	0.80	0.43459	0.39252	0.04207
**rs798443**	0.79	0.49985	0.48131	0.01854
**rs1462906**	0.78	0.42759	0.38318	0.04441
**rs3745099**	0.78	0.48537	0.45794	0.02743
**rs10007810**	0.77	0.501	0.42991	0.07109
**rs12913832**	0.77	0.35136	0.3785	-0.02714
**rs4908343**	0.77	0.49903	0.4486	0.05043
**rs772262**	0.77	0.47901	0.45327	0.02574
**MID406**	0.76	0.49763	0.48598	0.01165
**rs316598**	0.76	0.47901	0.41589	0.06312
**rs2593595**	0.75	0.4986	0.44712	0.05148
**rs6754311**	0.75	0.37115	0.3785	-0.00735
**MID1644**	0.74	0.45733	0.37559	0.08174
**rs9530435**	0.73	0.49319	0.40654	0.08665
**rs9522149**	0.73	0.49835	0.43662	0.06173
**rs13400937**	0.72	0.49518	0.47143	0.02375
**rs6451722**	0.72	0.49903	0.36449	0.13454
**rs9809104**	0.71	0.50028	0.46262	0.03766
**rs2986742**	0.71	0.49065	0.46262	0.02803
**rs4463276**	0.71	0.49837	0.48598	0.01239
**rs7803075**	0.71	0.48277	0.44393	0.03884

**rs874299**	0.71	0.49538	0.43458	0.0608
**rs1760921**	0.70	0.42759	0.42991	-0.00232
**rs2125345**	0.70	0.49319	0.5	-0.00681
**MID593**	0.70	0.43459	0.39252	0.04207
**rs6548616**	0.70	0.49871	0.49065	0.00806
**rs2196051**	0.69	0.49527	0.44545	0.04982
**rs7238445**	0.68	0.49932	0.44393	0.05539
**rs6464211**	0.68	0.48537	0.47664	0.00873
**rs385194**	0.67	0.48699	0.40187	0.08512
**MID3854**	0.67	0.40652	0.43458	-0.02806
**rs1503767**	0.67	0.47191	0.4455	0.02641
**rs2946788**	0.67	0.49932	0.45327	0.04605
**rs7251928**	0.67	0.50007	0.45283	0.04724
**MID2005**	0.67	0.48094	0.41589	0.06505
**rs10108270**	0.67	0.49932	0.44393	0.05539
**rs7421394**	0.67	0.49197	0.42523	0.06674
**rs1040404**	0.66	0.47901	0.46262	0.01639
**rs6556352**	0.66	0.49289	0.39378	0.09911
**rs1871428**	0.66	0.446	0.43458	0.01142
**rs2504853**	0.66	0.49377	0.48598	0.00779
**rs8113143**	0.66	0.48453	0.49065	-0.00612
**rs2238151**	0.66	0.48592	0.43868	0.04724
**rs4666200**	0.65	0.5009	0.47196	0.02894
**rs948028**	0.65	0.47572	0.46479	0.01093
**rs8021730**	0.63	0.49587	0.47664	0.01923
**rs260690**	0.63	0.46436	0.42056	0.0438
**rs1325502**	0.62	0.48366	0.45794	0.02572
**rs917115**	0.61	0.49763	0.42991	0.06772
**rs4984913**	0.61	0.5009	0.52804	-0.02714
**rs8035124**	0.61	0.46473	0.41622	0.04851
**rs9845457**	0.61	0.48277	0.46262	0.02015
**rs11227699**	0.61	0.38507	0.34112	0.04395
**rs37369**	0.61	0.45774	0.42523	0.03251
**rs7554936**	0.61	0.48925	0.47196	0.01729
**rs12544346**	0.60	0.46177	0.42056	0.04121
**rs1040045**	0.59	0.49985	0.48131	0.01854
**rs10839880**	0.59	0.49259	0.46729	0.0253
**rs4833103**	0.58	0.32584	0.30366	0.02218
**rs7844723**	0.58	0.4545	0.43192	0.02258
**MID3626**	0.58	0.49869	0.51643	-0.01774
**rs1296819**	0.58	0.49575	0.43575	0.06
**rs2330442**	0.58	0.47271	0.48131	-0.0086
**MID2011**	0.58	0.49634	0.48131	0.01503

**rs3784230**	0.57	0.45215	0.4218	0.03035
**rs200354**	0.57	0.49722	0.45327	0.04395
**rs2532060**	0.56	0.44285	0.47196	-0.02911
**rs1950993**	0.56	0.46588	0.47867	-0.01279
**rs6990312**	0.56	0.477	0.47196	0.00504
**MID881**	0.54	0.42759	0.38318	0.04441
**rs10236187**	0.54	0.41449	0.3785	0.03599
**MID943**	0.54	0.48777	0.46262	0.02515
**rs4821004**	0.53	0.49259	0.42991	0.06268
**MID2241**	0.52	0.50078	0.52336	-0.02258
**MID51**	0.52	0.48277	0.3972	0.08557
**MID1636**	0.51	0.48925	0.51869	-0.02944
**rs705308**	0.51	0.43961	0.42523	0.01438
**rs1500127**	0.51	0.37588	0.35047	0.02541
**MID798**	0.50	0.47901	0.43458	0.04443
**MID1802**	0.49	0.30467	0.28037	0.0243
**MID1726**	0.47	0.49985	0.44393	0.05592
**rs647325**	0.46	0.48277	0.50935	-0.02658
**MID1470**	0.45	0.47998	0.4486	0.03138
**MID2538**	0.44	0.41253	0.35514	0.05739
**rs2033111**	0.44	0.42323	0.38967	0.03356
**rs2835370**	0.44	0.3462	0.35047	-0.00427
**rs1837606**	0.41	0.50117	0.49533	0.00584
**MID128**	0.40	0.37353	0.36449	0.00904
**MID3122**	0.39	0.3258	0.31455	0.01125
**rs2166624**	0.39	0.38954	0.3972	-0.00766
**MID17**	0.38	0.4996	0.53271	-0.03311
**rs192655**	0.38	0.39538	0.37879	0.01659
**rs2627037**	0.38	0.3613	0.35849	0.00281
**rs4781011**	0.36	0.48619	0.50935	-0.02316
**rs1369093**	0.35	0.32578	0.25	0.07578
**MID2275**	0.35	0.3421	0.30516	0.03694
**MID1871**	0.33	0.29276	0.28972	0.00304
**MID659**	0.32	0.29577	0.2757	0.02007
**rs4458655**	0.31	0.39744	0.39336	0.00408
**rs2899826**	0.30	0.41055	0.35981	0.05074
**rs17642714**	0.29	0.2836	0.29439	-0.01079
**rs2024566**	0.28	0.39071	0.41784	-0.02713
**rs459920**	0.28	0.46045	0.5	-0.03955
**rs3943253**	0.27	0.31622	0.26168	0.05454
**rs7745461**	0.27	0.38731	0.37383	0.01348
**MID1603**	0.26	0.44068	0.42723	0.01345
**rs2070586**	0.26	0.41948	0.43662	-0.01714
**MID2256**	0.25	0.33676	0.28638	0.05038

**MID2264**	0.25	0.52788	0.50935	0.01853
**rs6541030**	0.24	0.29577	0.24766	0.04811
**rs1569175**	0.24	0.22305	0.215	0.00805
**MID360**	0.22	0.44526	0.43458	0.01068
**rs1513056**	0.22	0.4221	0.42523	-0.00313
**MID1607**	0.21	0.35895	0.38318	-0.02423
**MID548**	0.20	0.42395	0.41121	0.01274
**rs6104567**	0.20	0.36957	0.39698	-0.02741
**MID419**	0.20	0.32187	0.27103	0.05084
**rs3793791**	0.20	0.31049	0.30841	0.00208
**MID159**	0.20	0.46339	0.46009	0.0033
**rs818386**	0.20	0.19961	0.21495	-0.01534
**MID15**	0.19	0.47271	0.48131	-0.0086
**rs1471939**	0.19	0.42606	0.39267	0.03339
**rs2966849**	0.19	0.37821	0.36449	0.01372
**MID94**	0.18	0.29876	0.28972	0.00904
**rs4746136**	0.18	0.18567	0.16901	0.01666
**rs2030763**	0.17	0.24499	0.23832	0.00667
**rs174570**	0.15	0.27425	0.24299	0.03126
**rs734873**	0.14	0.18645	0.16981	0.01664
**rs1407434**	0.14	0.32414	0.30189	0.02225
**MID2313**	0.13	0.41055	0.42523	-0.01468
**rs731257**	0.13	0.24163	0.25234	-0.01071
**rs3118378**	0.13	0.47043	0.47196	-0.00153
**rs7722456**	0.12	0.39391	0.38785	0.00606
**MID1386**	0.11	0.30282	0.30516	-0.00234
**rs4717865**	0.11	0.13468	0.13551	-0.00083
**rs10512572**	0.11	0.34736	0.33333	0.01403
**rs12130799**	0.11	0.10611	0.1028	0.00331
**rs1879488**	0.10	0.29384	0.30986	-0.01602
**rs4951629**	0.10	0.28051	0.28037	0.00014
**rs12657828**	0.10	0.35136	0.29439	0.05697
**rs4918664**	0.10	0.15444	0.15888	-0.00444
**rs5768007**	0.10	0.22451	0.21028	0.01423
**rs7997709**	0.09	0.30383	0.28723	0.0166
**rs4955316**	0.09	0.24499	0.26636	-0.02137
**rs9319336**	0.09	0.23825	0.21963	0.01862
**rs2073821**	0.08	0.16984	0.14953	0.02031
**rs4670767**	0.08	0.17516	0.16022	0.01494
**MID777**	0.08	0.45635	0.46729	-0.01094
**MID250**	0.07	0.42023	0.3271	0.09313
**rs2357442**	0.07	0.25461	0.2891	-0.03449
**rs4880436**	0.07	0.13067	0.1215	0.00917

**MID196**	0.07	0.50064	0.5446	-0.04396
**MID2719**	0.07	0.48852	0.47664	0.01188
**rs3907047**	0.06	0.11026	0.11682	-0.00656
**rs10513300**	0.06	0.15444	0.15888	-0.00444
**rs1079597**	0.05	0.30172	0.30374	-0.00202
**MID1734**	0.05	0.3639	0.37383	-0.00993
**rs316873**	0.05	0.18861	0.20093	-0.01232
**rs12498138**	0.05	0.15985	0.15596	0.00389
**rs12439433**	0.05	0.08212	0.07487	0.00725
**rs2001907**	0.05	0.18269	0.18396	-0.00127
**rs214678**	0.05	0.20683	0.20561	0.00122
**rs946918**	0.04	0.36143	0.3972	-0.03577
**rs4411548**	0.04	0.32466	0.35047	-0.02581
**rs4798812**	0.04	0.40652	0.42523	-0.01871
**rs1513181**	0.04	0.34358	0.29907	0.04451
**rs32314**	0.04	0.41834	0.41589	0.00245
**rs2042762**	0.04	0.10611	0.09346	0.01265
**rs870347**	0.03	0.18117	0.18224	-0.00107
**rs10496971**	0.03	0.17364	0.16355	0.01009
**rs3737576**	0.03	0.15832	0.1729	-0.01458
**rs1834619**	0.03	0.13468	0.14486	-0.01018
**rs4918842**	0.02	0.34095	0.33178	0.00917
**MID2431**	0.02	0.27425	0.28037	-0.00612
**MID2929**	0.02	0.42023	0.42991	-0.00968
**rs3793451**	0.02	0.1849	0.19626	-0.01136
**rs2702414**	0.02	0.20683	0.19626	0.01057
**MID1193**	0.02	0.27739	0.28505	-0.00766
**rs4471745**	0.02	0.09217	0.08696	0.00521
**MID772**	0.02	0.20683	0.15888	0.04795
**rs1229984**	0.02	0.09395	0.09859	-0.00464
**rs10511828**	0.02	0.22102	0.19626	0.02476
**rs1876482**	0.01	0.10194	0.10748	-0.00554
**rs7226659**	0.01	0.13067	0.13084	-0.00017
**rs9291090**	0.01	0.09353	0.08879	0.00474
**MID397**	0.01	0.42759	0.39252	0.03507
**rs12629908**	0.01	0.23741	0.2451	-0.00769
**rs2306040**	0.00	0.14893	0.14141	0.00752
**rs3827760**	0.00	0.11849	0.12617	-0.00768
**rs881728**	0.00	0.24258	0.19718	0.0454
**rs671**	0.00	monomorphic		
**rs1800414**	0.00	monomorphic		
**rs3811801**	0.00	monomorphic		

Supplementary Table S3. Average ancestry proportion, range, and variance per component for the 214 unrelated Rio de Janeiro individuals reported by the five AIM sets.

		Average	Range	Variance
**46 indels**	**AFR**	0.3836	0.0490 - 0.9078	0.0461
	**EUR**	0.4560	0.0468 - 0.8700	0.0459
	**NAM**	0.1605	0.0404 - 0.4881	0.0082
**55 SNPs**	**AFR**	0.3841	0.0256 - 0.9602	0.0554
	**EUR**	0.5048	0.0180 - 0.9550	0.0552
	**NAM**	0.1111	0.0180 - 0.3868	0.0052
**122 SNPs**	**AFR**	0.3755	0.0208 - 0.9240	0.0537
	**EUR**	0.5486	0.0396 - 0.9568	0.0540
	**NAM**	0.0760	0.0116 - 0.4220	0.0043
**164 SNPs**	**AFR**	0.3834	0.0166 - 0.9566	0.0540
	**EUR**	0.5407	0.0230 - 0.9682	0.0540
	**NAM**	0.0759	0.0110 - 0.4057	0.0039
**210 AIMs**	**AFR**	0.3852	0.0160 - 0.9590	0.0528
	**EUR**	0.5401	0.0214 - 0.9720	0.0531
	**NAM**	0.0747	0.0110 - 0.3975	0.0038

Supplementary Table S4. Average ancestry proportions, range, and variance per component for the six American admixed populations reported by the five AIM sets.

ACB		Average	Range	Variance
46 indels	AFR	0.8759	0.3827 - 0.987	0.0114
	EUR	0.0758	0.007 - 0.4421	0.0063
	NAM	0.0483	0.006 - 0.3466	0.0035
55 SNPs	AFR	0.8784	0.5213 - 0.992	0.0090
	EUR	0.0931	0.004 - 0.4395	0.0080
	NAM	0.0285	0.004 - 0.1556	0.0007
122 SNPs	AFR	0.8886	0.5143 - 0.989	0.0074
	EUR	0.0908	0.0044 - 0.4709	0.0068
	NAM	0.0205	0.003 - 0.1758	0.0005
164 SNPs	AFR	0.8848	0.519 - 0.99	0.0071
	EUR	0.0984	0.005 - 0.4574	0.0064
	NAM	0.0167	0.003 - 0.1476	0.0003
210 AIMs	AFR	0.8825	0.525 - 0.9912	0.0073
	EUR	0.1000	0.0042 - 0.4404	0.0063
	NAM	0.0174	0.0026 - 0.166	0.0004

ASW		Average	Range	Variance
46 indels	AFR	0.7507	0.0240 - 0.9266	0.0319
	EUR	0.1594	0.0336 - 0.5725	0.0163
	NAM	0.0899	0.0154 - 0.5947	0.0102
55 SNPs	AFR	0.7689	0.0296 - 0.9550	0.0277
	EUR	0.1757	0.0170 - 0.4876	0.0125
	NAM	0.0554	0.0072 - 0.6669	0.0118
122 SNPs	AFR	0.7649	0.0552 - 0.9594	0.0251
	EUR	0.1857	0.0316 - 0.5214	0.0124
	NAM	0.0494	0.0058 - 0.5986	0.0113
164 SNPs	AFR	0.7652	0.0278 - 0.9484	0.0256
	EUR	0.1909	0.0366 - 0.5251	0.0112
	NAM	0.0440	0.0040 - 0.6290	0.0116
210 AIMs	AFR	0.7640	0.0170 - 0.9412	0.0261
	EUR	0.1924	0.0470 - 0.5428	0.0112
	NAM	0.0436	0.0040 - 0.6257	0.0115

CLM		Average	Range	Variance
**46 indels**	**AFR**	0.1639	0.0526 - 0.5344	0.0071
	**EUR**	0.5407	0.2150 - 0.8339	0.0192
	**NAM**	0.2954	0.0668 - 0.5921	0.0160
**55 SNPs**	**AFR**	0.1105	0.0268 - 0.4191	0.0077
	**EUR**	0.6299	0.2665 - 0.9152	0.0273
	**NAM**	0.2596	0.0438 - 0.5814	0.0158
**122 SNPs**	**AFR**	0.1101	0.0290 - 0.4477	0.0066
	**EUR**	0.6422	0.3067 - 0.9204	0.0181
	**NAM**	0.2477	0.0238 - 0.4828	0.0102
**164 SNPs**	**AFR**	0.0987	0.0188 - 0.4366	0.0068
	**EUR**	0.6476	0.3368 - 0.917	0.0195
	**NAM**	0.2536	0.0464 - 0.4919	0.0102
**210 AIMs**	**AFR**	0.0973	0.02 - 0.4049	0.0068
	**EUR**	0.6465	0.3405 - 0.904	0.0185
	**NAM**	0.2562	0.0474 - 0.4922	0.0099

PUR		Average	Range	Variance
**46 indels**	**AFR**	0.1914	0.0364 - 0.6881	0.0163
	**EUR**	0.6382	0.1834 - 0.8826	0.0221
	**NAM**	0.1703	0.0540 - 0.5048	0.0076
**55 SNPs**	**AFR**	0.1576	0.0274 - 0.7048	0.0124
	**EUR**	0.6554	0.1526 - 0.9152	0.0179
	**NAM**	0.1870	0.0378 - 0.4409	0.0077
**122 SNPs**	**AFR**	0.1584	0.0292 - 0.7512	0.0124
	**EUR**	0.7090	0.2164 - 0.8966	0.0139
	**NAM**	0.1327	0.0248 - 0.3465	0.0045
**164 SNPs**	**AFR**	0.1546	0.0364 - 0.7333	0.0119
	**EUR**	0.7006	0.2108 - 0.881	0.0135
	**NAM**	0.1448	0.022 - 0.3656	0.0039
**210 AIMs**	**AFR**	0.1566	0.0272 - 0.7301	0.0117
	**EUR**	0.7020	0.2204 - 0.8954	0.0133
	**NAM**	0.1414	0.0228 - 0.3261	0.0034

MXL		Average	Range	Variance
	AFR	0.0900	0.0308 - 0.2837	0.0034
46 indels	EUR	0.4414	0.0832 - 0.8702	0.0415
	NAM	0.4686	0.043 - 0.8604	0.0447
	AFR	0.0729	0.0172 - 0.192	0.0018
55 SNPs	EUR	0.4151	0.0288 - 0.8725	0.0388
	NAM	0.5120	0.0616 - 0.9444	0.0436
	AFR	0.0814	0.0152 - 0.1976	0.0024
122 SNPs	EUR	0.4382	0.0256 - 0.8626	0.0413
	NAM	0.4804	0.0504 - 0.9540	0.0422
	AFR	0.0649	0.0104 - 0.1616	0.0015
164 SNPs	EUR	0.4394	0.0182 - 0.8796	0.0402
	NAM	0.4956	0.0446 - 0.965	0.0418
	AFR	0.0572	0.0086 - 0.1504	0.0012
210 AIMs	EUR	0.4472	0.0258 - 0.8972	0.0395
	NAM	0.4956	0.032 - 0.9604	0.0419

PEL		Average	Range	Variance
	AFR	0.0588	0.0106 - 0.2936	0.0029
46 indels	EUR	0.2437	0.0230 - 0.7135	0.0321
	NAM	0.6975	0.1208 - 0.9640	0.0405
	AFR	0.0528	0.008 - 0.4859	0.0046
55 SNPs	EUR	0.1730	0.009 - 0.6455	0.0180
	NAM	0.7743	0.1562 - 0.983	0.0240
	AFR	0.0531	0.0068 - 0.4313	0.0041
122 SNPs	EUR	0.1553	0.0070 - 0.5397	0.0159
	NAM	0.7915	0.2410 - 0.9862	0.0228
	AFR	0.0471	0.005 - 0.4341	0.0040
164 SNPs	EUR	0.1685	0.0064 - 0.5842	0.0164
	NAM	0.7844	0.2025 - 0.987	0.0226
	AFR	0.0428	0.004 - 0.4115	0.0036
210 AIMs	EUR	0.1811	0.0068 - 0.5881	0.0164
	NAM	0.7761	0.2084 - 0.986	0.0229

Supplementary Table S5. Results obtained in the z-score analysis of the 65 sibling pairs from Rio de Janeiro. The percentages of sibling pairs rejected (both rejected) in African (AFR), European (EUR) or Native American (NAM) populations are indicated. For Rio de Janeiro, it is indicated the percentage of sibling pairs that were accepted (both accepted). For all populations, it is also indicated the percentage of siblings pairs with the same result, which means that both were rejected or both were accepted (same rate).

	AFR		EUR		NAM		RIO DE JANEIRO	
Panel	both rejected	same rate	both rejected	same rate	both rejected	same rate	both accepted	same rate
46 indels	96.92%	96.92%	55.38%	73.85%	100%	100%	81.54%	89.23%
55 SNPs	100%	100%	92.31%	95.38%	100%	100%	72.31%	80.00%
122 SNPs	100%	100%	83.08%	92.31%	100%	100%	75.38%	76.92%
164 SNPs	100%	100%	87.69%	92.31%	100%	100%	75.38%	81.54%
210 AIMs	100%	100%	89.23%	95.38%	100%	100%	67.69%	78.46%
